# Biomechanical and Neuromuscular Performance Requirements of Horizontal Deceleration: A Review with Implications for Random Intermittent Multi-Directional Sports

**DOI:** 10.1007/s40279-022-01693-0

**Published:** 2022-05-29

**Authors:** Damian J. Harper, Alistair J. McBurnie, Thomas Dos’ Santos, Ola Eriksrud, Martin Evans, Daniel D. Cohen, David Rhodes, Christopher Carling, John Kiely

**Affiliations:** 1 Institute of Coaching and Performance, School of Sport and Health Sciences, University of Central Lancashire, Fylde Road, Preston, PR1 2HE UK; 2 Department of Football Medicine and Science, Manchester United Football Club, AON Training Complex, Manchester, UK; 3 Department of Sport and Exercise Sciences, Musculoskeletal Science and Sports Medicine Research Centre, Manchester Metropolitan University, Manchester, UK; 4 Biomechanics Laboratory, Department of Physical Performance, Norwegian School of Sport Sciences, Oslo, Norway; 5 The FA Group, St George’s Park, Burton-Upon-Trent, Staffordshire, UK; 6 Faculty of Health Sciences, Masira Research Institute, University of Santander, Bucaramanga, Colombia; 7 Sports Science Centre (CCD), Colombian Ministry of Sport (Mindeporte), Bogotá, Distrito Capital Colombia; 8 Present Address: FFF Research Centre, French Football Federation, Clairefontaine National Football Centre, Clairefontaine-en-Yvelines, France; 9 Physical Education and Sports Science Department, University of Limerick, Limerick, Ireland; 10 Laboratory Sport, Expertise and Performance (EA 7370), French Institute of Sport (INSEP), Paris, France

## Abstract

Rapid horizontal accelerations and decelerations are crucial events enabling the changes of velocity and direction integral to sports involving random intermittent multi-directional movements. However, relative to horizontal acceleration, there have been considerably fewer scientific investigations into the biomechanical and neuromuscular demands of horizontal deceleration and the qualities underpinning horizontal deceleration performance. Accordingly, the aims of this review article are to: (1) conduct an evidence-based review of the biomechanical demands of horizontal deceleration and (2) identify biomechanical and neuromuscular performance determinants of horizontal deceleration, with the aim of outlining relevant performance implications for random intermittent multi-directional sports. We highlight that horizontal decelerations have a unique ground reaction force profile, characterised by high-impact peak forces and loading rates. The highest magnitude of these forces occurs during the early stance phase (< 50 ms) and is shown to be up to 2.7 times greater than those seen during the first steps of a maximal horizontal acceleration. As such, inability for either limb to tolerate these forces may result in a diminished ability to brake, subsequently reducing deceleration capacity, and increasing vulnerability to excessive forces that could heighten injury risk and severity of muscle damage. Two factors are highlighted as especially important for enhancing horizontal deceleration ability: (1) braking force control and (2) braking force attenuation. Whilst various eccentric strength qualities have been reported to be important for achieving these purposes, the potential importance of concentric, isometric and reactive strength, in addition to an enhanced technical ability to apply braking force is also highlighted. Last, the review provides recommended research directions to enhance future understanding of horizontal deceleration ability.

## Key Points

**Table:** 

Horizontal deceleration ability is defined as an athlete’s ability to proficiently reduce whole body momentum, within the constraints, and in accordance with the specific objectives of the task (i.e. braking force control), whilst skilfully attenuating and distributing the forces associated with braking (i.e. braking force attenuation).
During horizontal deceleration, braking steps exhibit a distinct ground reaction force profile characterised by high-impact peak forces and loading rates.
Horizontal deceleration ability is an adaptive coordinated outcome whereby neuromuscular and biomechanical qualities interact to optimise braking impulse and achieve desired reductions in whole body momentum.

## Introduction

Horizontal accelerations and decelerations are locomotor skills enabling athletes to execute rapid changes in velocity and direction, and are therefore crucial to sports requiring random intermittent multi-directional (RIMD) movement demands [1, 2]. As players perform frequent short-distance sprints during match play, horizontal acceleration ability is often regarded as the most critical skill for RIMD sport athletes [3, 4]. Accordingly, prior research has extensively examined the biomechanical and neuromuscular qualities underpinning superior horizontal acceleration ability in RIMD sport athletes [5–13], culminating in numerous evidence-informed guidelines on how to best monitor, train and coach this skill [4, 14–19].

In contrast, fewer studies have investigated the biomechanical and neuromuscular demands of horizontal deceleration. Subsequently, the potential factors underpinning superior horizontal deceleration performance are far less thoroughly understood. While deceleration has previously been described as the “forgotten factor” in sport-specific training [20], more recently, horizontal deceleration has nevertheless been shown to underpin rapid change of direction (COD) manoeuvres in athletes participating in RIMD sports [21–23]. Rapid horizontal deceleration ability also enables athletes to reduce momentum during very short time frames and distances to successfully evade or pursue opponents (i.e. to rapidly create and close down spaces) [24, 25]. Indeed, data derived from miniaturised wearable tracking technologies (i.e. global positioning systems) used ubiquitously in RIMD sports competition illustrate that high-intensity decelerations typically occur more frequently than equivalently intense accelerations in the majority of RIMD team sports [1]. For example, professional soccer players may perform 80–104% more high-intensity decelerations (*n* = 51–65), compared with accelerations (*n* = 27–35), during match play [26]. Accordingly, these actions have been shown to precede goals [27] and be an important feature associated with winning official matches in professional soccer [28]. Similar trends are reported in elite players in court-based team sports, such as basketball, to the extent that all positional roles perform significantly more high-intensity decelerations (3.2–4.5 per minute) than accelerations (0.8–1.5 per minute) during match play [29].

Although both accelerating and decelerating are regarded as the major biomechanical loading components in RIMD sports, they inevitably impose different physiological and mechanical demands [1, 30–34]. For example, during the single-leg support phase of stance, horizontal accelerations require a large propulsive impulse, with predominantly concentric muscle action (i.e. muscle shortening to do positive mechanical work) [34]. In contrast, horizontal decelerations impose a large braking impulse with predominant eccentric muscle action (i.e. muscle lengthening to absorb or do negative mechanical work) [34]. Importantly, in comparison to concentric muscle actions, eccentric muscle actions can generate higher mechanical forces [35]. Therefore, in comparison to accelerating rapidly, the braking steps associated with decelerating rapidly exhibit a unique ground reaction force (GRF) profile, characterised by a higher impact peak force and loading rate [30]. As such, intense decelerations when performed during match play typically impose ~ 41% greater mechanical load per meter than equivalently intense accelerations, and ~ 70% greater load per meter than other match play activities, such as high-speed running following an initial horizontal acceleration [36]. Accordingly, intense horizontal decelerations (< − 3 m·s^−2^) are a strong contributor to muscle damage in RIMD sports [37–40], and if not appropriately managed (i.e. acute spikes or cumulative high volumes of decelerations without sufficient recovery) may heighten the risk of incurring time-loss injury [41, 42]. Indeed, intense horizontal decelerations are one of the most common inciting events preceding non-contact anterior cruciate ligament (ACL) injury in RIMD sports [43–48], thus, further emphasising the importance of improving this skill and the ability to cope with the high mechanical demands it imposes.

Importantly, from the perspective of performance and ability to perform agile manoeuvres during match play, greater braking forces may enable decelerations to be achieved more rapidly in shorter time frames and distances, in comparison to accelerating rapidly [33, 49]. To illustrate, during soccer match play, players across all positional roles show higher magnitudes of decelerations (− 5.7 to − 6.3 m·s^−2^) than accelerations (4.4–4.7 m·s^−2^) [26]. Accordingly, rapid decelerations are vital to contemporary RIMD sports match play performance, especially as they create space that allows players greater time to execute technical skills and to transition into other high-intensity movement actions that facilitate successful offensive and defensive outcomes [1, 50]. Furthermore, evolutionary developments in RIMD sports including tactical team formations and game models (e.g. fast pressing and counterattacking playing styles) also suggest that these demands are likely to further increase in the future, meaning new insights into the biomechanical and neuromuscular demands of horizontal deceleration could have increasingly important considerations for RIMD sports performance and injury mitigation [51–54].

Accordingly, with the view of identifying implications for RIMD sports, the aims of this article were two-fold: (1) conduct an evidence-based review of the biomechanical demands of horizontal deceleration and (2) identify currently known biomechanical and neuromuscular performance (NMP) determinants. This review specifically targets implications for the future preparation of RIMD sport athletes for competition and promotes future research into the biomechanical and NMP requirements of horizontal deceleration.

## Literature Search Methodology

Original and review journal articles were retrieved from electronic searches of PubMed and MEDLINE (EBSCO) databases. Additionally, Google Scholar and bibliographic searches of relevant articles with no limits on year of publication were also completed. The search strategy included the following search terms and Boolean operators: “deceleration” AND “strength”, “deceleration” AND “biomechanics”, “deceleration AND “neuromuscular”, “deceleration” AND “change of direction”, “deceleration” AND “gait termination”, “deceleration” AND “turning”, “deceleration” AND “jump” and “deceleration” AND “braking”. The search concluded in November 2021. Only studies that measured deceleration kinetics or kinematics during straight-line horizontal acceleration-to-deceleration or severe COD tasks requiring substantial deceleration (≥ 90° turns) with physically active participants (no restrictions on age) were included [55, 56]. Furthermore, for COD studies to be eligible, they also had to include measurements during the preliminary deceleration steps that are necessary to reduce whole body momentum prior to the final foot contact of COD [57] or capture centre of mass (COM) horizontal velocity instantaneously throughout the COD task. The third and second to last foot contacts with the ground prior to the final foot contact of COD are referred to in the current article as the ante-penultimate and penultimate foot contacts, respectively [21].

## Biomechanical Demands of Horizontal Deceleration

The kinetic and kinematic demands of horizontal deceleration were previously compared to horizontal acceleration by Hewit and colleagues in 2011, although at this time, information was mostly qualitative and anecdotal [34]. More recent work has enhanced our understanding of the unique kinetic and kinematic demands of braking during rapid horizontal deceleration activities (Table 1). The next section discusses the biomechanical demands of horizontal deceleration using the sub-categories: (a) braking GRF, (b) whole body external mechanical forces and (c) braking force attenuation demands.

**Table 1:** Summary of biomechanical demands of horizontal deceleration and performance implications

Study	Participants	Deceleration task (measurement)	Biomechanical demand	Performance implications
Cesar and Sigward [85]	16 male physically active adults and 15 male children	Sprint to stop at 13-m known distance boundary (Qualisys 3D motion cameras, 250 Hz)	Children approach DEC at a higher percentage of their maximal velocity compared with adults (87 vs 77%) Adults have less anterior–posterior excursion of COM compared with children (48 vs 65 cm) during DEC Adults have greater relative posterior COM position during DEC compared with children (30 vs 14 cm) Adults have lower relative vertical COM position during DEC compared with children (86 vs 94 cm) Adults have lower percentage of time anterior to COM during DEC compared with children	Increased COM posterior position when decelerating will help to reduce anterior–posterior COM excursion Increased anterior COM position when decelerating may represent decreased stability and a more cautious deceleration strategy Quicker anticipatory postural adjustments and more effective braking force application in those more experienced with deceleration activities
Colby et al. [82]	15 collegiate and recreational athletes (9 were male and 6 were female)	Submaximal run to stop at 8-m known distance. EMG recorded on last step of stop (Pulnix 2D motion camera (60 Hz); Noraxon EMG, 600 Hz)	During last step of DEC, peak quadriceps activation was 161% of maximal seated isometric knee extension MVC and occurred at 39° of knee flexion (mid-stance) During last step of DEC, minimum hamstring activation was 39% of maximal seated isometric knee flexion MVC occurring at 28° of knee flexion (just after foot strike) Largest difference between quadriceps and hamstring activation during DEC occurred after minimal hamstring activation and just after peak quadriceps activation	During final step of DEC quadriceps activation is substantially greater than seated isometric knee extension MVC Increased quadriceps activation during DEC due to increased eccentric muscle action and passive elastic forces caused by high external GRF and joint flexion angular velocities upon ground contact Decreased hamstring activation in conjunction with increased quadriceps activation during DEC could increase chance of anterior tibial shear force that could increase ACL injury risk Increasing hamstring activation during DEC along with enhanced deceleration technique could reduce ACL injury risk
Dix et al. [103]	51 NCAA Division I and II female soccer players	10-m maximal ACC to DEC (Vicon motion cameras, 240 Hz; Bertec force plate, 1080 Hz)	Hip adduction angle (°): DL = 2.24, NDL = 1.06 Hip internal rotation angle (°): DL = 8.04, NDL = 8.41 Knee abduction angle (°): DL = − 2.00, NDL = − 1.32 Valgus collapse (°): DL = 3.02, NDL = 0.42 Hip adduction angle (°): Injured = 8.63, Uninjured = 1.66 Knee valgus collapse (°): Injured = 8.57, Uninjured = 0.65	Braking during DEC increases demand on proximal hip control to prevent hip adduction and knee valgus collapse Athletes who sustain future ACL injury may demonstrate increased hip adduction angle and knee valgus collapse during maximal DEC Maximal DEC could be used as a sport-specific screening task to identify athletes who may have increased risk of future ACL injury
Di Paulo et al. [98]	34 recreational and elite soccer players (18 were male and 16 were female)	10-m maximal ACC to DEC followed by backpedal (3D motion cameras, 120 Hz; Vicon motion cameras, 100 Hz; AMTI force plate, 120 Hz)	Average peak knee abduction moment during last step of horizontal DEC (Nm/kg): male player = 1.4, female player = 1.6 Peak knee abduction moment significantly higher in soccer players with high compared with low limb stability, frontal plane knee projection angle, GRF vector and total 2D video horizontal DEC movement score comprising: (1) limb stability, (2) pelvis stability, (3) trunk stability, (4) shock absorption and (5) movement strategy	2D video horizontal DEC movement score can identify players with high knee abduction moments during horizontal DEC and potential risk of knee injury, such as ACL 2D video horizontal DEC screening could evaluate movement quality during horizontal DEC and inform training and progression of this skill Knee joint overloading (i.e. peak knee abduction moment) most sensitive to frontal plane assessments (i.e. knee projection angle and GRF vector), highlighting importance of training exercises that can enhance lower-limb frontal plane control during rapid horizontal DEC
Dos’Santos et al. [62]	27 male multi-sport players (soccer *n* = 19, rugby *n* = 7, field hockey *n* = 1)	45, 90 and 180° COD with 5-m entry and 3-m to 5-m exit (Qualisys 3D motion cameras, 240 Hz; AMTI force plates, 1200 Hz)	FFC GCT (s): 45° = 0.20, 90° = 0.30, 180° = 0.51 Approach velocity (m·s^−1^): 45° = 5.22, 90° = 4.51, 180° = 4.00 Velocity at FFC (m·s^−1^): 45° = 5.06, 90° = 3.43, 180° = 2.68 Exit velocity (m·s ^−1^): 45° = 5.27, 90° = 3.29, 180° = 2.20 PFC peak horizontal GRF (BW): 45° = − 0.68, 90° = − 1.60, 180° = − 1.54 FFC peak knee abduction moment (Nm/kg): 45° = 0.83, 90° = 1.19, 180° = 0.85 FFC peak knee internal rotation moment (Nm/kg): 45° = − 0.50, 90° = − 1.00, 180° = − 0.48	Significantly greater GCT during FFC when there is a higher DEC demand prior to COD (i.e. slower stretch–shortening cycle demands in FFC) Higher braking forces during PFC of severe COD required to reduce momentum prior to FFC and facilitate faster COD If athletes cannot brake effectively in steps prior to FFC during sharper COD angles, it could expose athletes to higher knee joint loading during FFC, potentially increasing non-contact ACL injury risk Dual-foot contact braking strategy during 180° COD may help to decrease knee joint loads during FFC by more evenly distributing loads across both legs, in contrast to 90° COD, which appears to be more “high risk”
Falch et al. [92]	23 male Norwegian soccer players (2nd–6th league tier experience)	4 or 20-m maximal sprint with 45, 90, 135 or 180° COD and 4-m re-ACC (Xsens 3D inertial sensor motion-capture, 240 Hz)	Number of braking steps during DEC from 4 m approach: 45° = 0.4, 90° = 2.7, 135° = 3.3, 180° = 3.3 Number of braking steps during DEC from 20 m approach: 45° = 2.9, 90° = 5.4, 135° = 6.4, 180° = 6.3	‘Force-dominant’ COD (i.e. > 90°) involve significantly greater braking steps than velocity-dominant COD (i.e. < 90°) Increased approach distances (i.e. COM approach velocity) require more braking steps to reduce whole body momentum and distribute braking forces, highlighting the “multi-step” nature of DEC Increased approach velocities require greater braking forces to be generated in less time (i.e. increased braking impulse)
Gageler et al. [79]	3 recreational to professional running athletes	Sub-maximal self-selected run to DEC at 3 or 6-m known distance boundary (OptiTrack motion cameras, 100 Hz)	Mean peak trunk acceleration (g): 3 and 6-m DEC distance = ~ 6–7 g Mean peak ankle acceleration (g): 3-m DEC distance = ~ 25–27, 6-m DEC distance = ~ 18–24 Force attenuation ankle-to-knee (%): 3-m DEC distance = 76.5, 6-m DEC distance = 68.3 Force attenuation knee-to-sacrum (%): 3-m DEC = 10.8, 6-m DEC = 15.4 Force attenuation sacrum-to-upper torso (%): 3-m DEC distance = 12.7, 6-m DEC distance = 16	Ankle very high-impact forces during rapid DEC Ankle-to-knee muscle–tendon complexes attenuate most shock during each braking step of DEC Increased ankle-to-knee force attenuation demands with shorter enforced DEC Increased DEC distance enables force to be attenuated more evenly both between limbs and across steps Short rapid enforced DEC place greater force attenuation demands in early braking steps (i.e. steps 1–3 of 5) Increased frequency of rapid DEC could increase onset of fatigue and susceptibility to muscle–tendon damage and injury
Gray et al. [33]	10 male elite AFL players	Maximal DEC from high-speed run compared to maximal ACC (GPSports GPS receiver, 5 Hz)	Approach velocity prior to DEC (m·s^−1^) = ~ 8.00 Mean DEC time (s) = 2.1 Mean peak DEC (m·s^−2^) = − 5.3 Peak mechanical power (W/kg): DEC = 44, ACC = 31 Peak mechanical demand (J/kg.m): ACC = 6.8, DEC = 8.1 Total mechanical work during DEC (J/kg) = 58 (~ 52% DEC COM, ~ 32% swinging limbs, 14% vertical ACC/DEC of COM, 1% air resistance) Total mechanical work during ACC (J/kg) = 161 (~ 25% ACC COM, ~ 54% swinging limbs, 15% vertical ACC/DEC of COM, 6% air resistance)	Highest mechanical demand during DEC when the rate of change in velocity is greatest, i.e. peak DEC Intense DEC have a greater mechanical demand than ACC High peak mechanical demands when performing intense DEC could increase risk of tissue damage (i.e. mechanical and metabolic cost) DEC may require greater mechanical work than ACC to change COM velocity DEC may require less mechanical work than ACC to swing the limbs
Hader et al. [31]	12 male highly trained adolescent soccer players	10 or 15-m sprint, 90° turn and 10-m re-acceleration (Laveg laser guns, 100 Hz)	Peak DEC (m·s^−2^): 10 m = − 3.00, 15 m = − 3.29 DEC distance (m): 10 m = 7.1, 15 m = 8.7 No differences in quadriceps and hamstring EMG activity when approaching COD from greater approach distances and velocities Substantially greater quadriceps and hamstring EMG activity in most intense DEC phase of COD compared with horizontal sprint without COD Reduced metabolic power demands during DEC	Increased DEC with increased COD angle (i.e. angle-velocity trade-off) Decreased metabolic power when DEC due to increased eccentric muscle action and use of passive elastic structures Increased quadriceps and hamstring muscle activity during most intense DEC immediately prior to COD to support large external moments Quadriceps EMG greater than hamstrings during most intense DEC immediately prior to COD Increased quadriceps activity for attenuating high eccentric forces during most intense DEC phase Increased hamstring activity for knee stabilisation during most intense DEC phase Increased DEC distances prior to COD to maintain quadriceps and hamstring activity within neuromuscular capacities
Harper et al. [24]	38 university team sport players (29 were male and 9 were female)	20-m maximal ACC to DEC followed by backpedal (Stalker radar gun, 47 Hz)	Mean approach velocity (m·s^−2^) = 7.35 DEC distance-to-stop (m) = 6.86 DEC time-to-stop (s) = 1.50 Average deceleration (m·s^−2^) = − 4.44, early DEC phase = − 3.88, late DEC phase = − 5.99 Peak DEC (m·s^−2^) = − 8.48 Average HBP (W/kg) = − 17.43, early DEC phase = − 20.46, late DEC phase = − 12.58 Peak HBP (W/kg) = − 34.90 Average HBF (N/kg) = − 4.36, early DEC phase = − 3.66, late DEC phase = − 5.58 Peak HBF (N/kg) = − 8.44	Increased HBP in early DEC phase when COM velocity higher and time to apply force lower Increased peak DEC and HBF in late DEC phase when COM velocity lower and time to apply force higher Increased ability to brake during early DEC phase could reduce the magnitude of forces in late DEC phase and subsequent risk of tissue damage and injury
Havens and Sigward [77]	25 high-level soccer players (13 were male and 12 were female)	7.5-m sprint, 45 and 90° COD and 7.5-m re-acceleration (Qualisys 3D motion cameras, 250 Hz; AMTI force plates, 1500 Hz)	Mean approach velocity (m·s^−1^) = 4.72 Mean GCT (ms): PFC = 194, FFC = 252 Increased impulse, HBF and COM-COP distance during PFC compared to FFC in 90 vs 45° COD	Increase DEC demands during 90° vs 45° COD requiring increased foot to COM distance to generate posterior force Increased DEC demands requires increased impulse through increasing HBF and GCT Decreased perceptual and decision-making skills means reduced ability to pre-plan DEC strategy (i.e. anticipatory postural adjustments) in advance If lower extremity not prepared to deal with high external forces and moments during DEC, it could increase risk of passive tissue strains
Jones et al. [67]	26 female elite and sub-elite soccer players	10-m sprint, 90° COD and 3-m re-ACC (Qualisys 3D motion cameras, 240 Hz)	Increased peak HBF in PFC compared to FFC represents increased HBF ratio	Increase peak HBF during PFC could reduce knee abduction moments during FFC of COD, potentially modifying injury risk Increased peak HBF in PFC vs FFC represents increased HBF ratio HBF ratio provides information on braking strategy prior to COD
Jones et al. [64]	27 female second-tier English soccer players	10-m sprint, 180° COD and 5-m re-ACC (Qualisys 3D motion cameras, 240 Hz; AMTI force plates, 1500 Hz)	Mean approach velocity (m·s^−1^) = 4.02 Peak HBF (body weight): PFC = − 1.79, FFC = − 1.65	Increased peak HBF during PFC could reduce knee abduction moments during FFC, potentially reducing injury risk
Jones et al. [63]	22 female second-tier English soccer players	10-m sprint, with 90 or 180° COD and 3-m or 10-m re-ACC, respectively (Qualisys 3D motion cameras, 240 Hz; AMTI force plates, 1500 Hz)	Mean approach velocity (m·s^−1^): 90° COD = 4.40, 180° COD = 4.03 m·s^−1^ GCT (s): COD 90° PFC = 0.19, FFC = 0.26 GCT (s): COD 180° PFC = 0.38, FFC = 0.52 PFC = Peak knee external moments 30–40% of stance PFC = Increased plantar flexion external moment at 10% GCT, but increased dorsi flexion external moment at 50% GCT vs FFC 180° COD = increased peak vertical and HBF, knee and ankle flexion angles and average knee flexion and peak ankle PF moments during PFC vs FFC	Increased peak, but not mean HBF in PFC vs FFC Increased hip and knee flexor external moments must be counteracted in the PFC compared to FFC Increased braking force during PFC due to more upright and posterior trunk Increased HBF during PFC may reduce forces and injury risk during FFC Increased technical ability to apply braking forces prior to COD can increase COD performance Rapid dorsi and plantar flexion moments are required to accurately orientate, apply and attenuate braking forces
Jordan et al. [60]	1 male competitive soccer player	Maximal DEC following 20-m sprint performed at 50 (3.61 m·s^−1^), 75 (5.81 m·s^−1^) and 100% (6.45 m·s^−1^) self-perceived effort (Qualisys 3D motion cameras, 240 Hz)	GCT (s): 50% effort = 0.24, 75% effort = 0.18, 100% effort = 0.19 Foot-COM distance (m): 50% effort = 0.39, 75% effort = 0.40, 100% effort = 0.44 Dorsiflexion angular velocity (°/s^−1^): 50% effort = 229, 75% effort = 364, 100% effort = 377 Plantar flexion angular velocity (°/s^−1^): 50% effort = 307, 75% effort = 428, 100% effort = 484 Knee flexion angular velocity (°/s^−1^): 50% effort = 325, 75% effort = 461, 100% effort = 469 Hip flexion angular velocity (°/s^−1^): 50% effort = 90, 75% effort = 129, 100% effort = 168 Hip extension angular velocity (°/s^−1^): 50% effort = 74, 75% effort = 137, 100% effort = 121 Ankle ROM (°): 50% effort = 29, 75% effort = 31, 100% effort = 31 Knee ROM (°): 50% effort = 73, 75% effort = 79, 100% effort = 77 Hip ROM (°): 50% effort = 11, 75% effort = 9, 100% effort = 11	Plateauing effect on ankle and knee joint angular velocities when maximal DEC performed from sprint speeds between 75 and 100% maximal effort Ankle and knee very fast joint angular velocities, highlighting the importance of rapid limb switching in preparation for braking at ground contact Limited change in ankle, knee and hip ROM across entry speeds despite higher joint angular velocities, particularly ankle and knee (i.e. increased ankle and knee stiffness at higher approach speeds) Increased foot-COM distance when DEC from higher approach velocity to generate posterior braking force Longer foot GCT at slowest entry speed
Lozano-Berges [59]	12 recreational soccer players (9 were female and 3 were female)	4.5-m maximal ACC to DEC. GRF measured from first braking step with DL (Qualisys 3D motion cameras, 300 Hz; Kistler force platform, 3000 Hz)	Peak resultant GRF (N.kg) without/with cushioning underlay: horizontal braking step = 5.91/4.11, 90° cut plant step = 3.87/2.79, 180° cut plant step = 3.33/2.42, drop jump 30 cm = 2.73/1.70 Impulse (N.s.kg) without/with cushioning underlay: horizontal braking step = 0.14/0.10, 90° cut plant step = 0.11/0.08, 180° cut plant step = 0.07/0.06, drop jump 30 cm = 0.05/0.03 Loading rate (BW.s) without/with cushioning underlay: horizontal braking step = 466/329, 90° cut plant step = 329/219, 180° cut plant step = 192/149, drop jump 30 cm = 169/99	Braking during rapid horizontal deceleration most demanding task in terms of impact force characteristics Very high peak forces and loading rates during horizontal braking steps provides indication of impact severity and potential risk of overuse injuries Large interparticipant variability in magnitude of peak impact forces and loading rates highlights importance of interventions that can enhance horizontal DEC ability and thus reduce potential damaging consequences of exposure to repetitive high-impact mechanical forces Softer surfaces could help to mitigate exposure to high-impact forces when required to DEC frequently and could therefore help to reduce risk of overuse impact-related injuries. Implications also for progressive rehabilitation and for alleviating exposure to high forces in youth athletes
Mateus et al. [81]	14 elite male team sport players	Maximal ACC to DEC followed by backpedal. Estimation of forces and muscle contribution during last braking step (Qualisys 3D motion cameras, 500 Hz)	Muscles contributing to braking when DEC the COM = 1. Vasti ~ 76%, 2. Rectus femoris ~ 33%, 3. Soleus ~ 18% and gluteus maximus ~ 15% Muscles counteracting effect of gravity on COM when DEC = 1. Soleus ~ 54%, 2. Vasti ~ 44%, 3. Gluteus maximus = ~ 18%, 4. Rectus femoris = ~ 12% Muscles contributing to pull COM downwards during DEC = 1. Tibialis anterior = 21%, 2. Hamstrings = 15%	Quadriceps main contributor to COM DEC and for attenuating impact forces and supporting large external knee flexor moments when braking Hamstrings and soleus counteract quadriceps to create posterior shear force and prevent anterior translation of tibia Soleus important for increasing attenuation of impact when braking and for preventing forward sway by “locking the ankle” Gluteus maximus stabilizes trunk, eccentrically controls hip flexion and supports large hip flexor moment Tibialis anterior and hamstrings propel COM downwards and may therefore help with anterior foot placement precision when braking Lateral stabilisation (knee frontal plane control) when braking provided by gluteus medius counteracted by adductor magnus
Nedergaard et al. [61]	10 male soccer players	135° COD using dominant leg to turn (Qualisys 3D motion cameras, 500 Hz; Norxon accelerometer, 500 Hz)	Mean approach velocity (m·s^−1^) = 4.8 (~ 90% maximum) Mean peak trunk deceleration (g): APFC = 4.37, PFC = 4.58, FFC = 4.10 Mean GCT (ms): APFC = 193, PFC = 273, FFC = 471 Mean peak ankle joint angular velocity (°/s): PFC = 367, FFC = 255 Mean peak knee joint angular velocity (°/s): PFC = 493, FFC = 377	Preparatory braking steps prior to COD impose increased trunk deceleration and higher peak deceleration values than FFC step Reduced GCT during preparatory braking steps has significant effect on increasing peak trunk DEC Preparatory braking steps have increased ankle and knee joint angular velocities, representing increased loading severity and sensitivity to fatigue
Peel et al. [102]	11 recreationally active female	Sub-maximal paced run to reactive DEC to standstill (Vicon 3D motion cameras, 200 Hz)	Mean approach velocity = 4.5–5.0 m·s^−1^ Mean initial foot contact knee flexion angle = − 16° Peak knee abduction moment during first 10% of stance Peak anterior shear force = 9.51 N/kg^−1^ (~ 50% of stance) Peak internal knee extensor moment = 3.58 N m.kg^−1^ Anterior shear force in DEC correlated with anterior shear force in reactive cut (*ρ* = 0.67) Knee abduction moment in DEC small correlation with knee abduction moment in reactive cut (*ρ* = − 0.21)	Horizontal reactive DEC may have lower knee joint loads and ACL risk factors (anterior shear force and knee abduction moment) than reactive cut Anterior shear forces high during reactive DEC, but lower than in reactive 45° cut
Straub et al. [99]	39 intermittent sport players (15 were male and 24 were female)	4.6-m maximal ACC to DEC followed by backpedal (2D Simi Reality Systems motion cameras, 120 Hz; AMTI force plate, 1200 Hz)	Vertical GRF first peak at peak knee flexion (N.s/kg.m): 5.3 Vertical GRF impulse first peak at peak knee flexion (N.s^2^/kg.m): 0.16 2D sagittal plane thigh angle at peak knee flexion (°): 64.3 2D sagittal plane thigh angle significantly correlated with vertical GRF (R^2^ = 47%) and impulse first peak (R^2^ = 39%)	Increased 2D sagittal plane thigh angle (i.e. increased hip and knee flexion) can predict lower vertical GRF and impulse during last braking step of horizonal DEC. This highlights the importance of the ability to rapidly lower COM during horizontal DEC to reduce lower limb mechanical load 2D sagittal plane thigh angle can be used to characterise movement behaviour that may expose individuals to high-impact forces during horizontal DEC
Thomas et al. [66]	42 semi-professional soccer players (28 were male and 14 were female)	505_Tra_ COD test consisting of 15-m sprint, 180° turn and 5-m re-acceleration (Qualisys 3D motion cameras, 240 Hz; AMTI force plates, 1200 Hz)	Mean approach velocity (m·s^−1^): male = 5.2, female = 4.7 PFC DL mean peak HBF ratio: male = 1.05, female = 0.98 PFC NDL mean peak HBF ratio: male = 1.13, female = 0.97 PFC DL mean HBF ratio: males = 1.79, female = 2.14 PFC NDL mean HBF ratio: male = 1.96, female = 2.24 PFC DL peak hip flexion (°): male = 96, female = 86 PFC NDL peak hip flexion (°): male = 94, female = 79 Higher peak knee joint flexion angles and extensor moments during PFC than FFC Higher peak ankle dorsi flexion angles during PFC than FFC	Female individuals demonstrated an increased proportion of braking during FFC relative to PFC of COD compared with male individuals, which could increase loads to tolerate in FFC of COD, leading to increased ACL injury risk Male individuals demonstrated greater hip flexion during PFC, which could allow increased attenuation of forces through greater ROM Increased knee flexion and lower COM during preparatory DEC steps prior to COD could increase braking force application and preparation for the FFC of COD Increased dorsi flexion range during preparatory DEC steps may contribute to increased attenuation of forces and longer braking duration
Thomas et al. [68]	52 team sport players (24 were male and 28 were female)	505_Tra_ and 505_Mod_ COD test consisting of 15 or 5-m sprint, respectively, with 180° turn and 5-m re-acceleration (Qualisys 3D motion cameras, 240 Hz; AMTI force plates, 1200 Hz)	Greater peak hip and knee flexion angles in PFC than FFC Greater peak hip and knee extensor moments in PFC than FFC Greater ankle extensor moment in FFC than PFC Braking strategy influenced by leg asymmetry	Greater hip and knee flexion in PFC helps to lower COM to increase braking duration and braking impulse Greater hip and knee extensor moments in PFC required to control hip and knee flexion when braking during DEC Lower ankle extensor moment in PFC than FFC due to increased ankle dorsi flexion moment at initial heel strike when increased DEC demand Greater leg asymmetry may reduce DEC capacity prior to FFC of COD, potentially leading to increased braking forces and risk of injury in FFC
Verheul et al. [30, 58]	15 physically active team sport players (12 were male and 3 were female)	~ 20-m sprint to maximal DEC to standstill. GRF measured from first or second braking step (Kistler force platform, 3000 Hz)	Peak total GRF ~ 40–60 N/kg < 50 ms (~ 10–40% stance)	Rapid DEC braking steps have a distinct GRF profile characterised with high-impact peak and loading rates High-impact peak caused by forceful foot impact collision with the ground when braking, resulting in an increased segmental peak acceleration of braking leg foot, shank and thigh Abrupt segmental acceleration when braking increases contribution to whole-body biomechanical load
Zamparo et al. [32]	20 healthy Japanese sports players	5, 10, 15 or 20-m maximal shuttle run with 180° COD (Vicon 3D motion cameras, 100 Hz)	Mean approach velocity (m·s^−1^): 5 m = 4.22, 10 m = 5.45, 15 m = 6.21, 20 m = 6.75 DEC time (s): 5 m = 0.58, 10 m = 0.87, 15 m = 1.05, 20 m = 1.23 Average DEC (m·s^−2^): 5 m = − 6.82, 10 m = − 5.73, 15 m = − 5.06, 20 m = − 4.43 DEC mechanical power (W/kg^−1^): 5 m = 19, 10 m = 20, 15 m = 21, 20 m = 20 DEC/ACC mechanical power ratio: 5 m = 1.68, 10 m = 1.84, 15 m = 1.99, 20 m = 2.05 DEC/ACC ratio: 5 m = 1.74, 10 m = 1.75, 15 m = 1.77, 20 m = 1.75	Average external mechanical power between ~ 1.7 and 2 times greater in DEC vs ACC Increased mechanical power during DEC due to fundamental properties of eccentric muscle action Less time spent on DEC than ACC due to exploitation of increased eccentric muscle action and subsequent higher forces DEC to ACC ratio may provide useful information on individual ACC relative to DEC capacity. Low ratio indicates player has greater ACC capacity relative to DEC capacity. High ratio indicates player has high DEC relative to ACC capacity

*2D* two-dimensional, *3D* three-dimensional, *ACC* horizontal acceleration, *ACL* anterior cruciate ligament, *APFC* ante-penultimate foot contact, *COD* change of direction, *COM* centre of mass, *DEC* horizontal deceleration, *DL* dominant leg, *EMG* electromyography, *FFC* final foot contact, *GCT* ground contact time, *GRF* ground reaction force, *HBF* horizontal braking force, *HBP* horizontal braking power, *MVC* maximal voluntary contraction, *NDL* non-dominant leg, *PFC* penultimate foot contact, *ROM* range of movement

### Braking Ground Reaction Forces

**Fig. 1:**
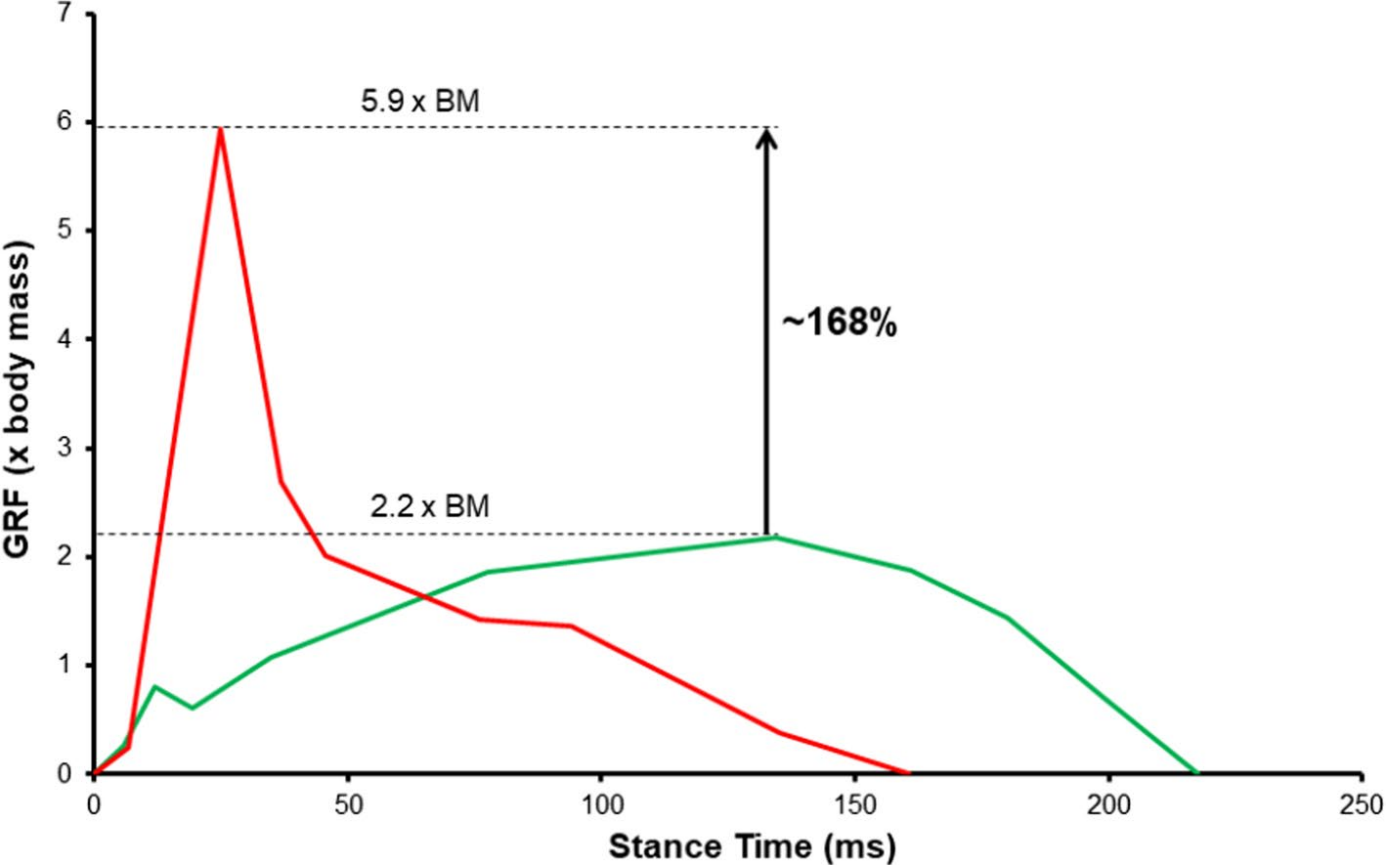
Comparison of ground reaction force (GRF) profiles during maximal horizontal deceleration (red line) and maximal horizontal acceleration (green line). Data taken from Verheul et al. [30]. *BM* body mass

During maximal horizontal decelerations, the magnitude of the rate of change in velocity has been reported to be ~ 17% greater than during maximal horizontal accelerations [49]. Likewise, during 180° COD tasks, average rates of change in velocity were 70–75% greater during the deceleration (− 4.43 to − 6.82 m·s^−2^) than the acceleration (2.55–4 m·s^−2^) phase, owing to much shorter time frames in which changes in velocity typically occur during rapid decelerations [32]. Consequently, when examining corresponding forces using Newtonian principles (i.e. force = mass × acceleration), the total exposure to GRFs are up to 2.7 times greater in magnitude during the initial steps of a maximal horizontal deceleration, than the corresponding steps of an acceleration [30]. For a 75-kg athlete, this would equate to impact forces of approximately 5.9 (58 N/kg) and 2.1 (21 N/kg) times the body mass during the first to second step of deceleration compared to acceleration [30]. As illustrated in Fig. 1, high-impact peak forces and loading rates (i.e. ‘tall-thin’ impulse) when braking during horizontal deceleration occur during the first 10–40% of the stance, and must be rapidly attenuated and distributed over very short time periods (< 50 ms) [30, 58, 59]. This is likely associated with the necessity for an anterior foot placement during intense horizontal decelerations, where an initial heel-strike of the ground would precede rapid plantar flexion of the foot [60, 61].

**Fig. 2:**
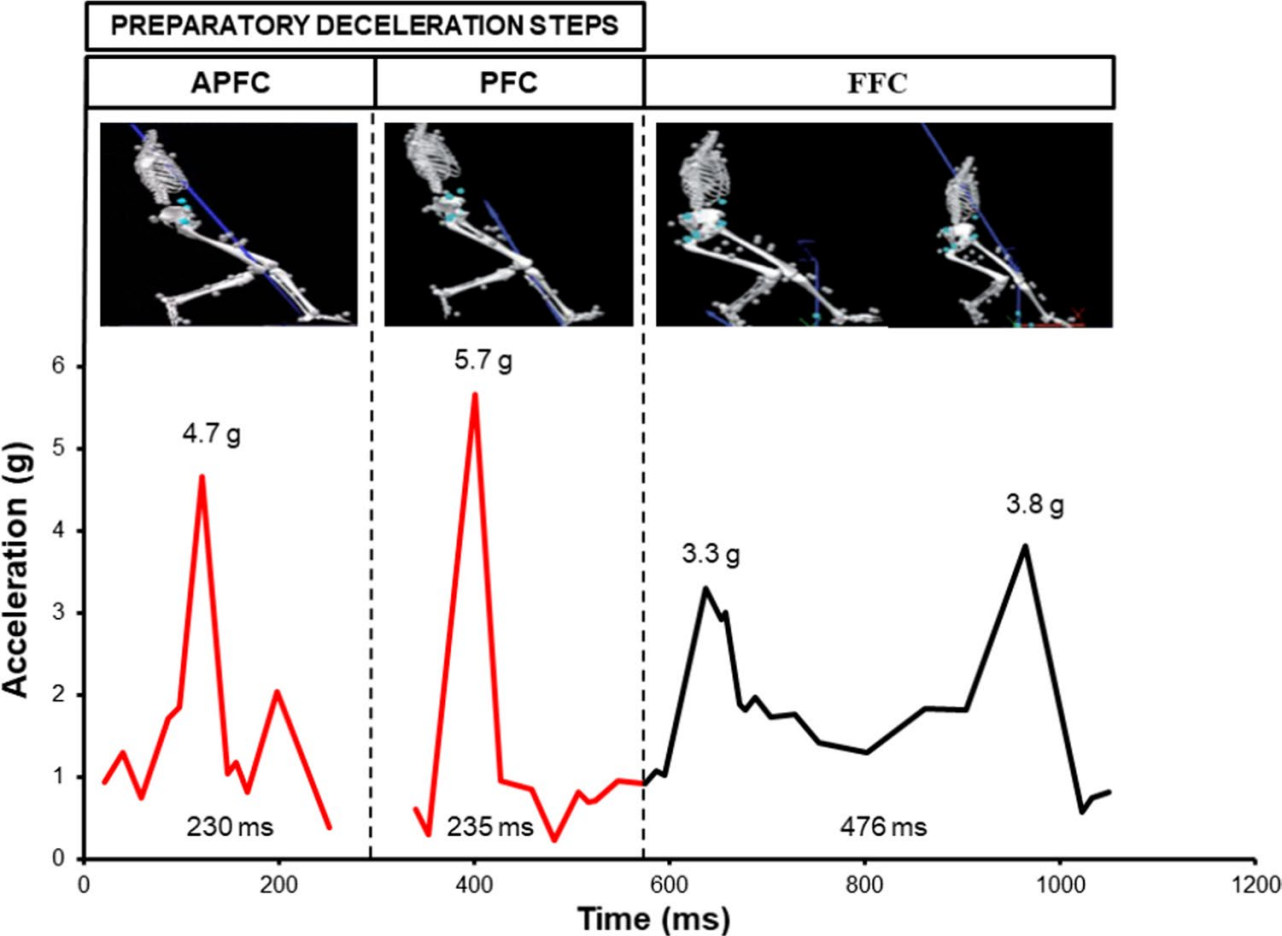
Trunk acceleration forces during ante-penultimate (APFC), penultimate (PFC) and final foot contact (FFC) of a severe 135° change of direction. Data taken from Nedergaard et al. [61]

During COD tasks demanding significant deceleration prior to turning (~ 135°–180°), similar GRF and trunk acceleration profiles have been reported during the preparatory deceleration steps (i.e. ante-penultimate and penultimate foot contact) prior to turning [21, 61]. In the study by Nedergaard et al. [61], the ante-penultimate and penultimate foot contact braking steps had ground contact times (GCT) of 230 and 235 ms, respectively, and comprised high-impact peak forces and loading rates (Fig. 2). Similarly, Dos’Santos et al. [21] reported mean GCTs of 199 ms in the ante-penultimate foot contact during a 180° COD, but the penultimate foot contact deceleration step was significantly longer (457 ms), perhaps because of players adopting a ‘dual-foot’ turning strategy (i.e. both feet in contact with the ground). Accordingly, an ability to produce greater deceleration in the steps prior to severe COD manoeuvres could be a deceleration strategy that not only enhances COD performance [21, 22, 62], but also reduces knee joint loads, which in turn could reduce ACL injury risk factors as they are commonly associated with turning during the final foot contact of COD manoeuvres [63–67].

It is also important to note that a player’s deceleration strategy could also be influenced by a lower-limb strength asymmetry or avoidance strategy, which manifests in a reduced ability of one limb to contribute to the generation and distribution of braking forces [22, 68, 69]. Consequently, one limb would disproportionately contribute to braking, thereby exposing this limb to greater mechanical loads, neuromuscular fatigue and injury risk, whilst also reducing deceleration and COD performance. Long-term exposure to asymmetrical braking forces, accompanied by insufficient recovery periods, subsequently acts to increase the potential for mechanical fatigue, thereby heightening the likelihood of major tissue failure and/or overuse injuries, such as tendonitis [70]. Accordingly, it would seem advantageous from a performance and injury mitigation perspective that athletes adopt a deceleration strategy that distributes braking forces proportionately and symmetrically between limbs. However, it should be acknowledged that asymmetrical loading patterns are commonplace in RIMD sports where a preferred limb is often used to predominantly perform game-based activities, such as kicking, jumping take-offs, and landings and the instigation of directional changes [71]. As such, each limb is exposed to differential magnitudes and rates and frequencies of mechanical forces, culminating in unique neuromechanical adaptations, especially in athletes with greater training and competition exposure [71]. As horizontal decelerations impose high mechanical forces, with an habitual loading pattern driving specific neuromuscular and structural adaptations, theoretically, performance would be lower and the injury risk elevated when forced to decelerate on the non-preferred less-frequently exposed limb. Accordingly, supplementary exercises should be prescribed to promote development of the neuromechanical qualities required to tolerate high braking forces, particularly for a player’s non-preferred braking limb.

### Whole Body External Mechanical Forces

Estimated whole body external mechanical forces generated during horizontal accelerations have been extensively investigated using running velocity data, leading to insights into player force–velocity capabilities and mechanical efficiency [72]. However, few studies have investigated the mechanical outputs associated with horizontal decelerations executed during intense sporting manoeuvres [24, 32, 33, 73]. During maximal horizontal deceleration, the average external mechanical power has been reported to be between ~ 1.7 and ~ 2 times greater than during the maximal acceleration phase of 5, 10, 15 and 20 m 180° COD tasks [32]. Similarly, the peak external mechanical power during a 2-s maximal horizontal deceleration is reported as ~ 1.7 times greater than that of a maximal horizontal acceleration recorded across the same time period (~ − 44 vs 26 W/kg, respectively) [33]. Harper et al. [24] reported similar high external peak horizontal braking power (− 35 W/kg) outputs during a maximal horizontal deceleration performed from average horizontal sprint velocities of 7.35 m·s^−1^. Such high mechanical power outputs, observed during rapid horizontal decelerations, are likely due to the accentuated eccentric work, whereby skeletal muscle properties facilitate higher forces owing to stretching of passive tissue structures [74].

When examining different phases of horizontal deceleration, the highest average horizontal braking power outputs have been reported to occur during the early (peak velocity [*V*_max_] to 50% *V*_max_), compared to the late (50% *V*_max_ to minimum velocity) horizontal deceleration phase (20 vs 13 W/kg) [24]. Similarly, in the study by Gray et al. [33], peak mechanical power also occurred during the early horizontal deceleration phase (at ~ 1 s of a 2.4-s deceleration) of an intense deceleration event in team sport training, which was just prior to the peak deceleration. Additionally, the peak mechanical demand was reported to coincide with the peak deceleration during the early horizontal deceleration phase and was 16% greater than that measured during a maximal horizontal acceleration (8.1 vs 6.8 J/kg·min^−1^, respectively) during a 40-m horizontal sprint. It is important to note that, in the study by Gray et al. [33], mechanical power and mechanical demand were modelled from total mechanical work calculated from velocity–time data captured using a global positioning satellite device. As such, it may not be surprising that the highest horizontal mechanical power occurred during the early deceleration phase, as this is the period when COM velocity is highest. Unlike mechanical power, the highest average horizontal braking forces have been reported to occur during the late deceleration phase (− 5.60 vs − 3.66 N/kg, respectively) [24]. Collectively, these findings highlight the importance of generating higher braking forces during the early deceleration phase, when momentum is higher and the time to apply force is shorter [21]. Indeed, higher horizontal braking power outputs have been observed in female team sport athletes with both faster 45° and 180° COD times, presumably owing to higher forces being generated with less GCT at higher approach velocities [75].

An initial braking period, during which only slight reductions in COM velocity occur, has previously been associated with preparatory postural adjustments required to stabilise COM and enhance braking force application [76, 77]. Subsequently, a longer braking preparation time, in the early deceleration phase, is likely associated with greater deceleration demands and higher braking forces during the late deceleration phase [78]. Alternatively, the longer preparatory braking period could be driven by a deceleration strategy allowing COM velocity to be slightly reduced, and dynamic stability to be maintained up to a hypothetical ‘critical’ threshold (i.e. self-regulated based on perceived physical capacity by the player). Beyond this ‘critical’ threshold, the eccentric forces encountered when braking exceed the load bearing and braking force capacities of the player, highlighting the potential ‘performance-injury conflict’ often observed when performing intense COD manoeuvres [55].

### Braking Force Attenuation Demands

Analysis of enforced horizontal decelerations during sub-maximal runs has shown that the ankle and knee muscle–tendon structures attenuate between 68 and 77% of the impact forces emanating from foot ground contact [79]. In the same study, when decelerations were more intense, the demands on the ankle and knee muscle–tendon structures to attenuate forces further increased, with the earlier braking steps (i.e. step 1 of 5) imposing greater peak segmental acceleration and shock attenuation demands. Conversely, when decelerations were less intense, impact forces were of lower magnitude and distributed more evenly between limbs and across more steps. Aligning with this, Verheul et al. [58] identified that the GRF impact peak when braking during a rapid deceleration was caused by high peak segmental accelerations of the braking foot, shank and thigh. Accordingly, peak segmental accelerations experienced when braking hard make a substantial contribution to whole-body biomechanical load in RIMD sports with a high frequency of rapid decelerations. Therefore, performing a higher frequency of rapid decelerations (i.e. repetitive braking cycles) during match play likely increases the onset of fatigue and the risk of soft-tissue damage if the muscle–tendon structures are not adequately recovered or optimally prepared for these demands [70]. Theoretically, these risks are likely to be further exacerbated following detraining of specific eccentric NMP qualities [80] or during fixture congestion when players experience less recovery time between games [70].

During intense horizontal decelerations when the COM is positioned posterior to the base of support, the knee extensors counteract the large external knee flexor moments [55] and are key contributors to attenuating the high-impact braking forces arising upon ground contact [81]. Indeed, the vasti muscles demonstrate a high level of pre-activation prior to ground contact and, when required to attenuate eccentric forces during ground contact, electromyograph values can exceed 150% isometric maximal voluntary contraction [82]. As an indication of these extreme loading demands, values for knee joint angular velocities have been reported between 469 and 493°/s during the braking steps of an intense horizontal deceleration [60] and during the penultimate foot contact of deceleration prior to COD [61]. Such high knee joint angular velocities highlight the importance of knee extensor pre-activation and fast eccentric force production capabilities. Accordingly, during rapid deceleration, a high internal knee extensor moment is required to safely control and attenuate forces across relevant knee joint flexion ranges, particularly during the early phases of braking when high-impact forces and loading rates may be experienced [63, 68].

The biarticular rectus femoris is a key quadriceps muscle responsible for attenuating eccentric forces during deceleration [81], especially when the trunk assumes a more erect posture [83]. When the trunk adopts a more forward lean in order to reduce quadriceps forces [82], help lowering and control of COM [84], or when forward trunk excursion cannot be controlled because of high deceleration rates (i.e. sling shot effect) [85], the gluteus maximus and hamstrings will likely contribute to controlling external hip flexion moment and to attenuating and distributing forces [31, 81]. Indeed, in competitive matches, rapid horizontal decelerations during defensive pressing actions are one of the major situational patterns commonly associated with major lower extremity injuries, such as ACL rupture [43, 45, 47, 48, 86, 87]. In these studies, a lack of trunk control, lowering of COM (i.e. extended knee at initial contact), and lateral foot placement when decelerating are often observed as the major biomechanical causes of injury. The soleus has also been identified as an important muscle contributing to the attenuation of impact forces during deceleration, in addition to resisting the vertical effect of gravity and preventing forward sway by “locking the ankle” [81]. It is also important to note that in addition to the hamstrings, the soleus also exerts a posterior shear force that counteracts anterior translation of the tibia that could decrease ACL strain and injury risk when decelerating rapidly [81]. Therefore, although quadriceps strength is clearly crucial for enhancing horizontal deceleration, other muscle groups meaningfully contribute to horizontal deceleration management capabilities [88]. For example, increasing maximal eccentric hamstring strength may help reduce the decline in hamstring rate of force development (RFD) and neuromuscular function (i.e. electromechanical delay) following fatiguing exercise, which could be critical for maintaining neuromuscular control and stability of the knee joint during rapid horizontal decelerations [89]. Similarly, hamstring fatigue resistance has been shown to be enhanced following a 7-week strength training intervention focusing on developing hamstring strength-endurance in female RIMD sport athletes [90].

Increasing the capacity of the muscle–tendon unit to withstand high eccentric braking forces logically serves to enhance deceleration ability and mitigate injury risk [83]. For example, when performing a deceleration from two different COD approach speeds (6.40 vs 7.11 ms^−1^), no differences in vastus lateralis and biceps femoris muscle activation profiles were observed, despite differences in forward momentums at each speed [31]. Instead, when approaching from the higher speed (7.11 ms^−1^) players began decelerating earlier, and subsequently decelerated over longer distances, perhaps to maintain forces within what they perceive they can safely tolerate. This observation implies the influence of self-regulatory protective mechanisms and the “multi-step” nature of intense horizontal deceleration challenges [91, 92]. Consequently, if these capacities are improved through training, it is plausible to conclude that deceleration distance will be reduced, and overall COD performance time improved, but this remains to be investigated.

## Biomechanical Determinants of Horizontal Deceleration

A summary of research studies that have investigated the biomechanical determinants of horizontal deceleration ability are included within Table 2. We discuss these determinants in the forthcoming section and include braking force technical application and braking GRF magnitude.

**Table 2:** Summary of studies investigating biomechanical determinants of horizontal deceleration ability

Study	Subjects	Deceleration task	Biomechanical determinants of deceleration	Magnitude of determinant	Performance implications
Cesar and Sigward [95]	16 male adults and 15 male physically active children	Sprint to stop at 13-m known distance boundary (Qualisys 3D motion cameras, 250 Hz)	Increased COM posterior position	*R*^2^ = 46–52%	Increased COM posterior position increases whole-body stability during DEC and posterior force generation Increased DEC demand requires increased COM posterior position If DEC demand greater than strength capacity, COM position adjusted to control magnitude of braking force through having less posterior and higher vertical COM position Different braking strategies in individuals with greater approach momentum prior to DEC
			Increased COM posterior position and lower COM vertical position in children	*R*^2^ = 52–64%	
Dos’ Santos et al. [22]	40 male sub-elite rugby league and collegiate team sport players	505_Mod_ COD test (AMTI force plates 1200 Hz)	Peak HBF (N/kg) in PFC (right leg FFC): fast = 12.4, slow = 12.4	ES = 0.01 (T)	Greater HBF with reduced GCT (i.e. tall-thin impulse) in PFC associated with faster COD performance Lower HBF ratio means greater momentum reduced prior to COD facilitating faster COD performance Increased inter-limb asymmetry in HBF may reduce COD performance and increase injury risk
			Peak HBF (N/kg) in PFC (left leg FFC): fast = 14.2, slow = 11.3	ES = 1.08 (M)	
			PFC GCT (s) (right leg FFC): fast = 0.35, slow = 0.39	ES = − 0.68 (M)	
			PFC GCT (s) (left leg FFC): fast COD 0.39, slow COD 0.44	ES = − 2.43 (VL)	
			HBF ratio (right leg FFC): fast = 1.11, slow = 1.20	ES = − 0.31 (S)	
			HBF ratio (left leg FFC): fast = 0.82, slow = 1.19	ES = − 1.50* (L)	
Dos’Santos et al. [84]	61 male team sport athletes	505_Tra_ and 505_Mod_ COD test (Qualisys 3D motion cameras (240 Hz); AMTI force plates 1200 Hz)	PFC H-VBF mean ratio 505_Mod_: fast = − 0.70 slow = − 0.61	ES = − 1.72 (L)	Increased technical ability to apply HBF associated with more rapid DEC prior to COD and faster COD performance times Greater PFC hip, knee and ankle flexion angles increases COM lowering, facilitating greater technical ability to generate high braking impulse (i.e. more horizontal braking and duration) Forward trunk inclination could help increase lowering of COM during DEC Increased braking capacity prior to COD enables higher approach velocities to be achieved prior to COD Increased braking likely due to increased eccentric strength Importance of braking late and rapidly for faster COD times
			PFC H-VBF peak ratio 505_Mod_: fast = − 0.71, slow = − 0.59	ES = − 1.43 (L)	
			PFC RBF angle (°) of peak 505_Mod_: fast = − 55, slow = − 59	ES = 1.48 (L)	
			PFC peak hip flexion angle (°) 505_Mod_: fast = 100, slow = 81	ES = − 1.56 (L)	
			PFC peak knee flexion angle (°) 505_Mod_: fast = 119, slow = 106	ES = 1.34 (L)	
			PFC H-VBF mean ratio 505_Tra_: fast = − 0.73, slow = − 0.60	ES = − 2.41 (VL)	
			PFC H-VBF peak ratio 505_Tra_: fast = − 0.74, slow = − 0.65	ES = − 1.13 (M)	
			PFC RBF angle (°) of peak 505_Tra_: fast = − 54, slow = − 57	ES = 1.35 (L)	
			PFC peak hip flexion angle (°) 505_Tra_: fast = 97, slow = 81	ES = − 1.24 (L)	
			PFC peak knee flexion angle (°) 505_Tra_: fast = 118, slow = 104	ES = 1.31 (L)	
			Δ PFC-FFC velocity (m·s^−1^) 505_Tra_: fast = − 3.71, slow = − 3.47	ES = − 0.64 (M)	
Dos Santos et al. [21]	20 male university soccer players	505_Tra_ COD test (AMTI force plates 1200 Hz)	APFC angle (°) of peak RBF to 505 time	*r* = 0.74 (VL)	Greater HBF and impulse application during DEC increases ability to rapidly reduce COM velocity and momentum APFC pivotal role in braking prior to COD Increased importance on preparatory deceleration steps when approaching COD from higher sprint velocities Better technical ability to apply HBF associated with faster DEC and COD performance To improve a player’s 180° COD ability, coaches should look to develop players' ability to brake rapidly (magnitude and orientation of braking force) from varied sprint velocities, whilst coaching a multi-step strategy
			APFC peak H-VBF ratio to 505 time	*r* = − 0.74 (VL)	
			APFC mean H-VBF ratio to 505 time	*r* = − 0.78 (VL)	
			APFC peak HGRF to 505 time	*r* = − 0.63 (L)	
			APFC mean HGRF to 505 time	*r* = − 0.74 (VL)	
			APFC peak RBF to 505 time	r = − 0.52 (L)	
			APFC mean RGRF to 505 time	r = − 0.64 (L)	
			APFC horizontal total impulse	*r* = − 0.53 (L)	
			PFC mean H-VGRF ratio to 505 time	*r* = − 0.57 (L)	
			PFC peak H-VBF ratio to 505 time	*r* = − 0.49 (M)	
			PFC angle (°) of peak RBF to 505 time	*r* = − 0.48 (M)	
Falch et al. [75]	25 female team sport players (16 handball and 9 soccer)	20-m maximal ACC to DEC followed by backpedal (MUSCLELAB Laser)	ACC power during 20-m ACC-to-DEC test (W/kg): 8.39		Significantly greater HBP in the fast 180° COD group despite lower HBF than the slow 180° COD group is indicative of faster production of net GRF to reduce momentum and less time spent braking Moderate to large differences in HBF and HBP, respectively, in fast compared to slow 45° COD highlight ability to reduce braking time and maintain velocity throughout the COD
			HBP during 20-m ACC-to-DEC test: − 10.51		
			HBP to ACC power ratio: 1.25		
			HBP (W/kg): fast 180° COD = − 11.58, slow 180° COD = − 9.73	ES = 1.34 (L)	
			HBF (N/kg): fast 180° COD = − 2.88, slow 180° COD = − 3.34	ES = 0.09 (T)	
			HBP (W/kg): fast 45° COD = − 11.33, slow 45° COD = − 9.49	ES = 1.44 (L)	
			HBF (N/kg): fast 45° COD = − 3.27, slow 45° COD = − 2.81	ES = 1.07 (M)	
Kaneko et al. [23]	70 male youth soccer players	505_Tra_ COD test (Laveg, LDM 300C Sport laser, 100 Hz)	Peak DEC (m·s^−2^): fast COD = − 11.02, slow = − 9.60	ES = -0.98 (M)	Increased peak DEC prior to COD associated with better overall COD performance Poorer DEC ability associated with higher peak DEC closer to COD turn line potentially meaning more time spent during transition to re-acceleration and higher injury risk Fast COD group had higher DEC potential 1 m prior to turn line, resulting in a lower approach velocity that may facilitate less time turning and quicker transition to re-acceleration
			Position of peak DEC (m·s^−2^): fast COD = 4.53 m, slow = 4.78 m	ES = − 1.13 (M)	
			DEC 1 m prior to COD (m·s^−2^): fast COD = − 7.71, slow = − 6.14	ES = − 1.15 (M)	
			DEC significant association with total 505 COD speed time	*P* = 0.041	
Santoro et al. [97]	40 college basketball players (32 were male and 8 were female)	505_Mod_ COD test (AMTI force plates 2400 Hz, 5-m Optojump)	*505*_*Mod*_* descriptive data* Average 505_Mod_ completion time (s): 2.77 Turning phase duration (s): 1.22 (44% of 505_Mod_ completion time) Total number of steps (*n*): 8–10 Velocity at last foot contact prior to turn (m·s^−1^): 5.40		Players with faster COD can generate higher braking forces and impulses in PFC to enable DEC from faster approach velocities PFC characterised with a single braking phase, highlighting the importance of eccentric strength to enhance DEC and COD performance Players with faster COD have longer step lengths and ground contact times in PFC, indicating greater anterior foot placement relative to COM and the importance of this braking step in positioning body for re-acceleration
			*Players with braking/propulsive forces in first re-acceleration step*		
			Approach velocity (m·s^−1^): fast = 5.72, slow = 5.13	ES = 1.51 (L)	
			PFC step length (cm): fast = 126, slow = 92	ES = 0.92 (M)	
			Braking horizontal impulse (N.s/kg): fast = 1.5, slow = 1.2	ES = 1.3 (L)	
			PFC ground contact time (s): fast = 0.392, slow = 0.355	ES = 0.48 (S)	
			*Players with propulsive-only forces in first re-acceleration step*		
			PFC step length (cm): fast = 123, slow = 98	0.75 (M)	
			Braking horizontal GRF (N/kg): fast = 16.7, slow = 12.9	1.06 (M)	
			PFC ground contact time (s): fast = 0.403, slow = 0.352	0.58 (M)	

*3D* three-dimensional, *505*_*Mod*_ modified 505 change of direction test with 5-m approach distance, *505*_*Tra*_ traditional 505 change of direction test with 15-m approach distance, *ACC* horizontal acceleration, *APFC* ante-penultimate foot contact, *COD* change of direction, *COM* centre of mass, *DEC* horizontal deceleration, *ES* effect size [interpreted as: *T* trivial (0–0.19), *S* small (0.20–0.59), *M* moderate (0.60–1.19), *L* large (1.20–1.99), *VL* very large (2.0–4.0)], *r* correlation [interpreted as: *M* moderate (0.30–0.49), *L* large (0.50–0.69), *VL* very large (0.70–0.89)], *FFC* final foot contact, *GCT* ground contact time, *HBF* horizontal braking force, *HBP* horizontal braking power, *H-VBF* horizontal-to-vertical braking force, *H-VGRF* horizontal-to-vertical ground reaction force, *PFC* penultimate foot contract, *RBF* resultant braking force, *RGRF* resultant ground reaction force

### Technical Ability

When performing a horizontal deceleration manoeuvre, there is a complex sequence of muscle activation and de-activation strategies to enable precise intra-limb and inter-limb coordination to optimise the effectiveness of force application when braking [93]. Similar to horizontal acceleration [94], the technical ability to apply a more horizontally orientated GRF vector is important for facilitating more effective braking and for achieving more rapid horizontal deceleration ability [21, 84]. Lower vertical and more posterior COM positions relative to the lead leg braking foot are important postural positions to maintain dynamic stabilisation and facilitate application of a more horizontally orientated braking force [95]. These braking postures result in less anterior COM excursion when braking, helping to maintain COM position behind the lead limb braking foot, thereby prolonging the time in which horizontal braking forces can be applied (i.e. impulse-momentum relationship) [85]. Indeed, players who are able to perform more rapid deceleration during severe COD tasks have greater hip, knee and ankle dorsi-flexion angles (i.e. triple flexion), allowing them to dynamically lower COM position to ensure more horizontal component of the GRF when braking [84].

Whilst not previously considered in sports performance research, the ability to dynamically lower and stabilise the COM behind the lead limb braking foot during rapid decelerations is also reliant upon the coordinated actions of the trailing limb [96]. Indeed, during rapid deceleration the tibialis anterior and hamstrings are the main flexors at the ankle and knee joints, respectively, helping to lower COM and maintain it behind the lead limb braking foot [81]. Furthermore, strong activation of the tibialis anterior acts to inhibit soleus plantar flexor propulsive impulses in the trailing limb that help to prolong COM posterior position and braking force application of the leading limb [96].

### Braking Ground Reaction Force Magnitude

In addition to possessing a greater technical ability to apply a more horizontally orientated braking force, RIMD sport players with faster COD performance times also generate greater peak horizontal braking forces in the penultimate foot contact prior to COD than those with slower COD performance times [22]. This braking strategy reduces horizontal momentum prior to the final foot contact and is suggested to facilitate more effective weight acceptance and push-off propulsive forces during the final foot contact of COD, contributing to faster COD performance [22]. However, it is important to note that this braking strategy was not evident across both limbs (i.e. turning directions). This again highlights the importance of ensuring braking force capabilities are developed across both limbs, to reduce potential multi-directional performance deficits and injury risk resulting from inadequate braking force production prior to COD, and when required from less favourable turning directions.

In accordance with the technical ability to apply forces in a more horizontally orientated direction, faster deceleration and COD performance times have also been reported in RIMD sport athletes who can produce greater peak and mean magnitude of forces in the horizontal versus vertical direction (i.e. ratio of forces) in both the ante-penultimate and penultimate foot contact steps of COD [21, 84]. These findings align with the impulse-momentum relationship, which signifies that changes in horizontal deceleration are proportional to the directional magnitude of the force applied. When comparing the influence of the ante-penultimate and penultimate foot contacts on COD performance times, the ante-penultimate foot contact has been reported to have a much more substantial role [21]. This is likely owing to braking being performed in the sagittal plane, where more optimal postural positions can be adopted to facilitate the application of a greater magnitude of horizontal braking force, and subsequent deceleration of momentum prior to COD. For example, the magnitude of the mean horizontal and horizontal-to-vertical GRF ratio generated during the ante-penultimate foot contact explained between 55 and 61% of COD performance time, highlighting the importance of being able to generate high horizontal braking forces to enhance both deceleration and COD performance. In contrast, the penultimate foot contact, in a 180° COD manoeuvre (i.e. 505 test), could be viewed primarily as a ‘positional’ deceleration step to facilitate weight acceptance and ‘drive-off’ during the final foot contact [21, 97]. It is important to acknowledge that braking strategies adopted in a traditional 505 test may not accurately reflect those seen during actual match play, when braking and turning manoeuvres occur spontaneously in response to dynamically unfolding events. Accordingly, future research is needed to substantiate these findings across different COD and agility (i.e. with less preparatory time) tasks, and to investigate the GRF profiles across the whole deceleration phase of COD and their associations with deceleration, COD performance and lower limb mechanical loading. Furthermore, as the preparatory deceleration steps that have a greater impact on COD performance seem to occur in the sagittal plane, future research should investigate if deceleration capabilities measured during a COD task are associated with performance in that task only, or also with the deceleration capabilities in tasks without a COD. Such findings could lend further support for the use of horizontal acceleration-to-deceleration tasks without a COD for: (1) profiling a player’s ability to decelerate rapidly prior to COD, (2) identifying athletes at a heightened risk of injury because of high-impact forces and knee joint loads [98, 99] and (3) helping to determine when an athlete is ready to return to sport following injury [100–103].

## Neuromuscular Determinants of Horizontal Deceleration Ability

Previously, although anecdotal, the four major NMP determinants of deceleration were suggested as: (1) eccentric strength, (2) reactive strength, (3) power and (4) dynamic balance [20]. Table 3 provides a summary of studies that have investigated the NMP determinants of deceleration ability, including eccentric, reactive, and concentric strength qualities and RFD.

**Table 3:** Summary of studies investigating neuromuscular performance determinants of horizontal deceleration ability

Study	Subjects	Deceleration task (measurement)	Neuromuscular performance determinants of horizontal deceleration	Magnitude of determinant	Deceleration performance implications
Greig and Naylor [111]	19 male university team sport players	10-m maximal ACC to reactive DEC to stop (tape measure)	Mean reactive DEC DTS (m) = 5.59		Greater eccentric hamstring strength associated with faster reactive DEC ability Greater eccentric hamstring strength required to increase hip extensor torque, control trunk and knee flexion when braking during reactive DEC Greater ability to maintain eccentric hamstring strength at higher joint angular velocities associated with better reactive DEC Greater concentric quadriceps strength at higher joint angular velocities associated with better reactive DEC
			ECC KF PT 60°/s and reactive DEC DTS	*r*^2^ = 32%	
			ECC KF PT 60°/s, ECC KF PT fast (180°/s): slow (60°/s) ratio and reactive DEC DTS	*r*^2^ = 53%	
			ECC KF PT 60°/s, ECC KF PT fast (180°/s): slow (60°/s) ratio, ECC KF angle of PT 60°/s and reactive DEC DTS	*r*^2^ = 62%	
			ECC KF PT 60°/s, ECC KF PT fast (180°/s): slow (60°/s) ratio, ECC KF angle of PT 60°/s, CON KE PT 180°/s and reactive DEC DTS	*r*^2^ = 70%	
Graham-Smith et al. [25]	9 S&C coaches	Maximal ACC-to-DEC stopping at 5, 10, 15 or 20 m (Laveg Laser, 100 Hz)	Average DEC gradient (m·s^−1^ per m): − 0.74 (range: − 0.55 to − 0.90)		Greater eccentric quadriceps and hamstring strength associated with better DEC ability Low shared variance (21–28%) between eccentric quadriceps strength and DEC ability highlights importance of other factors, such as technical ability to apply braking force
			% V_max_: 5 m = 54, 10 m = 72, 15 m = 83, 20 m = 89		
			DEC DTS (m): 5 m = 2.9, 10 m = 4.9, 15 m = 6.6, 20 m = 7.9		
			ECC KE PT 60°/s and average DEC gradient	*r* = − 0.53 (L)	
			ECC KF PT 60°/s and average DEC gradient	*r* = − 0.47 (M)	
Harper et al. [107]	14 male English academy soccer players	20-m maximal ACC to DEC followed by backpedal (2D digital camera, 50 Hz)	ECC KE PT at 60°/s DL and DEC TTS and DTS	*r* = − 0.54 to − 0.63 (L)	Greater unilateral eccentric quadriceps strength at slower joint angular velocities associated with better DEC ability (i.e. distance and time to stop) Greater unilateral concentric quadriceps and hamstring strength at faster knee joint angular velocities associated with better DEC ability (i.e. less distance and time to stop)
			ECC KE PT at 60°/s NDL and DEC TTS and DTS	*r* = − 0.55 to − 0.64 (L)	
			CON KE PT at 180°/s DL and DEC TTS and DTS	*r* = − 0.54 to − 0.55 (L)	
			CON KE PT at 180°/s NDL and DEC TTS and DTS	*r* = − 0.64 to − 0.76 (L-VL)	
			CON KF PT at 180°/s DL and DEC DTS	*r* = − 0.54 (L)	
			CON KF PT at 180°/s NDL and DEC TTS and DTS	*r* = − 0.61 to − 0.78 (L-VL)	
Harper et al. [73]	27 male university team sport players	20-m maximal ACC to DEC followed by backpedal (Stalker Radar, 47 Hz)	CMJ CON peak force (N/kg^−1^): high DEC = 26, low DEC = 24	ES = 0.95 (L)	Players with better DEC ability can generate higher concentric forces during CMJ Players with better DEC ability can generate higher eccentric peak forces during DEC phase of CMJ Players who can generate higher horizontal braking impulse (i.e. reduce momentum faster) can generate higher eccentric and concentric peak velocities during CMJ than players with low horizontal braking impulse
			CMJ CON mean force (N/kg^−1^): high DEC = 20, low DEC = 19	ES = 0.91 (L)	
			CMJ ECC peak force (N/kg^−1^): high DEC = 25, low DEC = 23	ES = 0.72 (M)	
			CMJ ECC-DEC RFD (N.s/kg^−1^): high DEC = 99, low DEC = 81	ES = 0.58 (M)	
			CMJ CON peak velocity (m·s^−1^): high HBI = 2.8, low HBI = 2.5	ES = 1.15 (L)	
			CMJ ECC peak velocity (m·s^−1^): high HBI = − 1.3, low HBI: − 1.1	ES = − 1.00 (L)	
Harper et al. [78]	29 university team sport players (23 were male and 6 were female)	20-m maximal ACC to DEC followed by backpedal (Stalker Radar (47 Hz)	DJ20 and DJ40 RSI and average DEC	*r* = − 0.61 (L)	Players with greater DJ-RSI demonstrate superior DEC ability Greater DJ concentric mean force associated with better DEC ability; however, DJ40 eccentric peak force significantly associated with DJ concentric mean force Greater DJ-RSI associated with better early DEC ability, meaning these players can brake quicker in the initial steps of DEC Players who can brake early have better overall DEC ability
			DJ20 and DJ40 CON mean force and average DEC	*r* = − 0.65 to − 0.67 (L)	
			DJ20 and DJ40 RSI and early DEC phase	*r* = − 0.62 to − 0.65 (L)	
			DJ20 and DJ40 CON mean force and early DEC phase	*r* = − 0.54 to − 0.66 (L)	
			DJ40 ECC mean force and DJ20 and DJ40 CON mean force	*r* = 0.64 to 0.77 (L-VL)	
			Average early DEC phase and average DEC	*r* = 0.93 (AP)	
Jones et al. [91]	18 female soccer players	505_Tra_ COD test (Qualisys 3D motion camera, 240 Hz)	Mean approach velocity = 3.88 m·s^−1^		Players with greater eccentric quadriceps strength can DEC more rapidly in PFC prior to COD, permitting faster approach velocities Players with greater eccentric quadriceps strength can approach COD at higher velocities because of ability to generate higher braking forces Greater eccentric quadriceps strength associated with ability to generate higher peak and mean horizontal braking forces, enabling more rapid DEC prior to COD
			ECC KE PT 60°/s and approach velocity	*r* = 0.72 (VL)	
			ECC KE PT and Δ in velocity PFC	*r* = − 0.56 (L)	
			ECC KE PT and Δ in velocity PFC to FFC	*r* = − 0.44 (M)	
			ECC KE PT and Δ in velocity PFC: strong = − 1.55, weak = − 1.37	ES = − 0.94 (M)	
			ECC KE PT and peak PFC HBF: strong = − 2.16, weak = − 1.77	ES = − 1.0 (M)	
			ECC KE PT and mean PFC HBF: strong = − 0.53, weak = − 0.45	ES = − 1.2 (L)	
			ECC KE PT and PFC peak hip extensor moment: strong = − 3.57, weak = − 2.90	ES = − 0.95 (M)	
Zhang et al. [108]	14 French national female soccer players	20-m maximal ACC to DEC followed by backpedal (Stalker Radar, 47 Hz)	ECC KE PT at 30°/s NDL and average HBF	*r* = − 0.71 (VL)	Maximal unilateral ECC quadriceps torque at slower joint angular velocities in NDL has strongest association with average horizontal force, power and impulse during a rapid DEC, demonstrating importance of training interventions to enhance this quality Ability to generate quadriceps ECC torque rapidly in NDL important for enhancing horizontal force, power and impulse during rapid DEC Concentric quadriceps PT in NDL at slower joint angular velocities associated with greater horizontal braking force and impulse during rapid DEC Concentric quadriceps PT in DL and NDL at faster joint angular velocities associated with greater maximal HBP during rapid DEC Ability to generate high rates of torque development in CON quadriceps and hamstrings potentially important for lower limb stiffness, dynamic knee joint control and force attenuation during rapid DEC
			CON KE PT at 60°/s NDL and average HBF	*r* = − 0.54 (L)	
			ECC KE RTD_100_ DL and average HBF	*r* = − 0.54 (L)	
			ECC KE PT at 30°/s NDL and average HBP	*r* = − 0.70 (VL)	
			ECC KE RTD_100_ DL and average HBP	*r* = − 0.63 (L)	
			CON KE PT at 60°/s NDL and average HBI	*r* = − 0.55 (L)	
			ECC KE PT at 30°/s NDL and average HBI	*r* = − 0.68 (L)	
			ECC KE RTD_100_ DL and average HBI	*r* = − 0.54 (L)	
			ECC KE PT at 30°/s NDL and maximum HBF	*r* = − 0.61 (L)	
			CON KE PT at 240°/s DL and maximum HBP	*r* = − 0.57 (L)	
			CON KE PT at 240°/s NDL and maximum HBP	*r* = − 0.58 (L)	
			CON KF PT at 240°/s DL and maximum HBP	*r* = − 0.58 (L)	
			RTD_100_ KF CON/KE CON DL ratio and maximum HBP	*r* = − 0.61 (L)	
			RTD_100_ KF CON/KE CON NDL ratio and maximum HBP	*r* = − 0.59 (L)	
			ECC KE PT at 30°/s NDL and maximum HBI	*r* = − 0.75 (VL)	
			RTD_100_ KF CON/KE CON DL ratio and maximum HBI	*r* = − 0.57 (L)	

*2D* two-dimensional, *3D* three-dimensional, *505*_*Tra*_ traditional 505 change of direction test with 15-m approach distance, *ACC* horizontal acceleration, *AP* almost perfect (0.90–0.99), *CON* concentric, *DEC* horizontal deceleration, *DJ* drop jump, *DTS* distance-to-stop, *ECC* eccentric, *ES* effect size [interpreted as: *T* trivial (0–0.19), *M* moderate (0.60–1.19), *L* large (1.20–1.99), *VL* very large (2.0–4.0)], *GRF* ground reaction force, *HBF* horizontal braking force, *HBI* horizontal braking impulse, *HBP* horizontal braking power, *KE* knee extensor, *KF* knee flexor, *PFC* penultimate foot contact, *PT* peak torque, *r* correlation [interpreted as: *M* moderate (0.30–0.49), *L* large (0.50–0.69), *VL* very large (0.70–0.89)], *r*^2^ = coefficient of determination, *RSI* reactive strength index, *RTD* rate of torque development, *TTS* time to stop, *V*_*max*_ peak velocity

### Eccentric Strength Qualities

**Fig. 3:**
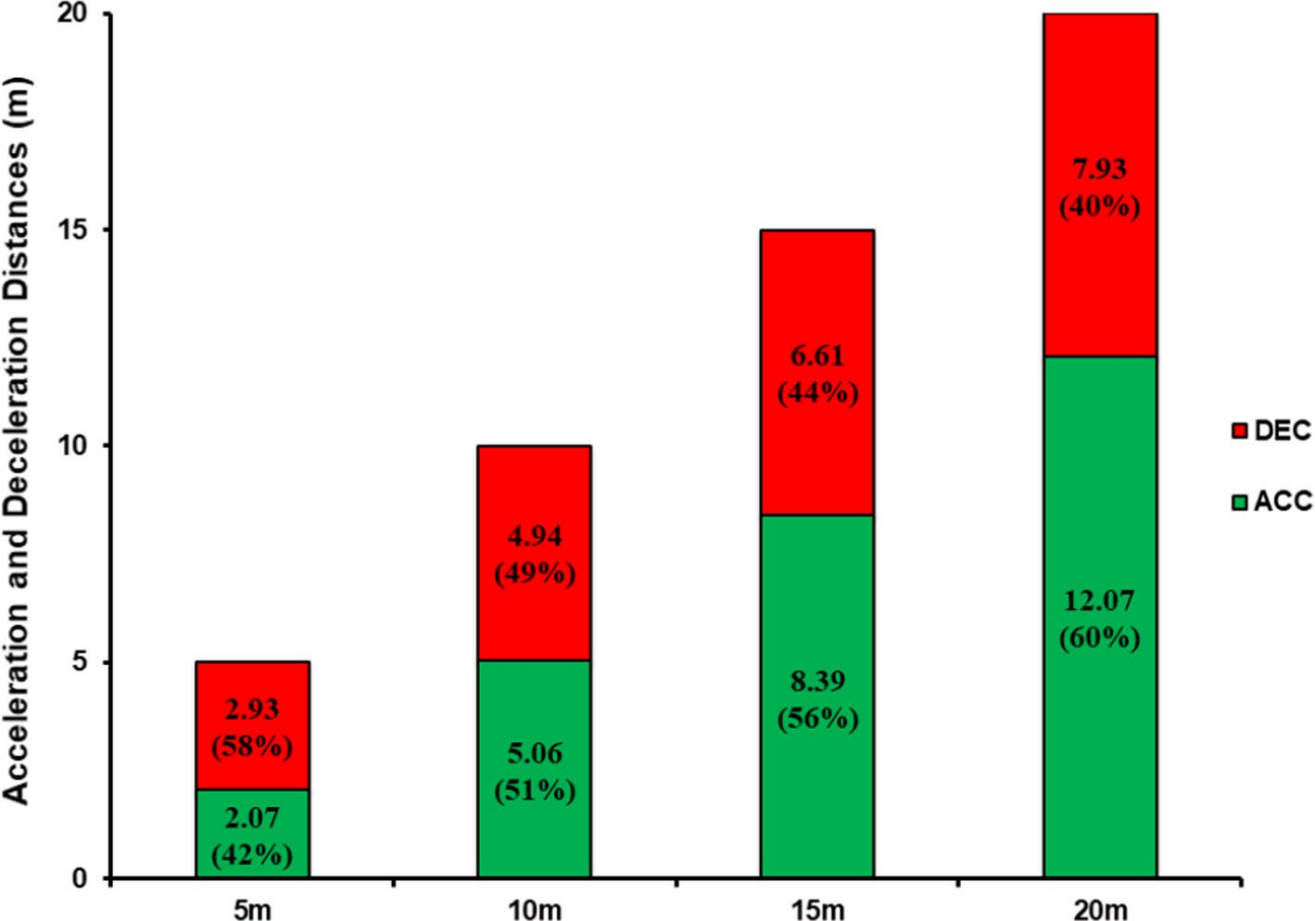
Distance spent accelerating (ACC) and decelerating (DEC) from different sprint-to-stop distance trials (percentage time is illustrated in brackets). Data from Graham-Smith et al. [25]

The importance of eccentric muscle strength for enhancing rapid horizontal deceleration ability has been widely acknowledged in reviews on COD, for both performance enhancement [55, 57, 104] and injury risk reduction [100, 101, 105, 106]. Indeed, a number of studies have demonstrated moderate to very large associations between unilateral isokinetic eccentric knee extensor (quadriceps) strength measured at slower joint angular velocities (i.e. 30–60°/s) and horizontal deceleration ability [25, 91, 107, 108]. In addition to maximal unilateral eccentric quadriceps strength, large associations have also been reported between rapid (0–100 ms) eccentric quadriceps torque and horizontal braking force, power and impulse during a rapid horizontal deceleration [108]. The authors suggested that the ability to generate rapid eccentric quadriceps torque increases passive torque rise and the contribution of passive tissue structures to the generation and management of high GRF when braking during maximal horizontal deceleration manoeuvres. A comparison of players with high and low eccentric quadriceps strength showed that those with superior eccentric strength had a significantly greater ability to generate horizontal braking forces [91]. Consequently, these players were able to approach COD at higher speeds because of being able to produce and tolerate greater braking forces, contributing to both quicker decelerations prior to COD and faster overall COD performance times. Furthermore, in the study by Graham-Smith et al. [25], greater eccentric quadriceps and hamstring strength contributed to better horizontal deceleration ability, measured using the ‘deceleration gradient’, an indicator of how much speed per metre a player could decelerate. Interestingly, this study also highlighted the substantial distances spent decelerating across the various sprint-to-stop activities most commonly encountered by RIMD sport athletes during match play. Deceleration distances varied between 2.39 and 7.93 m for 5-m and 20-m sprint-to-stop distance trials, distances equivalent to between 58 and 40%, respectively, of the total distance covered (Fig. 3). Although the deceleration distance to stop is likely to vary between different athletic populations with different physical capacities and individual horizontal deceleration abilities, these findings demonstrate the importance of horizontal deceleration, in addition to horizontal acceleration, to RIMD sports performance outcomes. Thus, the competitive advantage for players with enhanced braking capabilities might be a reduction in their deceleration distance and time to stop, allowing them to attain a greater percentage of their maximal sprinting speed prior to decelerating by having more time and distance to accelerate prior to deceleration.

Eccentric peak force and eccentric-deceleration RFD, measured during vertical countermovement jumps (CMJs), have also been reported to be significantly greater in players with high compared with low horizontal deceleration ability when quantified using average deceleration (m·s^−2^) from a maximal horizontal deceleration following a maximal 20-m sprint [73]. Both eccentric peak force and eccentric-deceleration RFD occur during the downwards phase of a CMJ when knee joint flexion velocities, of around 133–199°/s, need to be decelerated prior to transitioning into the upward phase of the jump [109]. Accordingly, these CMJ metrics may be indicative of similar NMP qualities required to decelerate horizontal momentum prior to a severe COD task, providing a good proxy for horizontal deceleration ability. Additionally, these findings highlight the importance of evaluating and training various eccentric NMP qualities for enhancing maximal horizontal deceleration ability. For example, fast eccentric loading exercises could be deployed to enhance eccentric-deceleration RFD [110].

An additional potentially important research finding is the lower association between eccentric hamstring strength (isokinetic knee flexion) and horizontal deceleration, than between eccentric quadriceps (isokinetic knee extension) strength and horizontal deceleration performance [25, 91, 107, 108]. However, as the muscle group also contributes to hip extensor movement during horizontal deceleration [68], future research should also assess associations between hip extensor strength and horizontal deceleration ability. Nonetheless, higher quadriceps than hamstring activation is observed during horizontal deceleration activity [31, 82], presumably necessary to counteract high external knee flexion moments and to facilitate the generation of the high braking impulses required to quickly reduce momentum. It has also been suggested that lower associations may be due to the muscle group functioning primarily isometrically when braking during horizontal deceleration [108]. Subsequently, these authors highlighted the importance of future research investigating hamstring function during horizontal deceleration manoeuvres. It is also possible that increased eccentric hamstring force generation could be more associated with unanticipated horizontal deceleration, as opposed to pre-planned manoeuvres, owing to an increasing demand for eccentric hamstring force generation for trunk and pelvic control and rapid knee joint stabilisation [111].

### Reactive Strength Qualities

Reactive strength has previously been proposed to be an important NMP determinant of deceleration ability [20]. However, only one prior study by Harper et al. [78] has investigated the associations between this quality and maximal horizontal deceleration ability using drop jump (DJ) reactive strength index (RSI; jump height/GCT) measured from both 20-cm and 40-cm drop heights. Both had large significant correlations with average horizontal deceleration ability, and it is noteworthy that they also had significant large correlations with average horizontal deceleration during the early deceleration phase (i.e. *V*_max_ to 50% *V*_max_), but not with the late deceleration phase (i.e. 50% *V*_max_ to minimum velocity). These findings suggest that greater DJ-RSI scores, particularly from higher drop heights, are likely representative of a player’s ability to rapidly adjust posture and generate greater braking impulses during the earlier deceleration phase when much shorter time frames to generate forces are available. Furthermore, DJ-RSI measured from similar drop heights (~ 50 cm) has also been reported to have significant large correlations (*r* = 0.60, *R*^2^ = 36%) with maximal isoinertial eccentric squat strength performed at a controlled descent of ~ 30°/s [112]. Indeed, only one longitudinal study to date has examined the influence of enforced decelerations on various isokinetic knee strength capacities [113]. Here, only the group that included enforced decelerations demonstrated a significant increase in DJ 40 cm-RSI and maximal eccentric isokinetic hamstring strength, highlighting the potential stimulus of this training modality for lower limb eccentric strength development. Together, these findings confirm the importance of lower limb reactive and eccentric maximal strength as NMP determinants of rapid horizontal deceleration ability. Consequently, training interventions that can enhance reactive and eccentric strength, and the underpinning NMP qualities of reactive strength, seem to be important considerations for enhancing horizontal deceleration ability.

### Concentric Strength Qualities

Whilst not previously considered as a determinant of rapid deceleration ability, concentric strength capabilities have also been identified as potentially important NMP qualities contributing to rapid horizontal deceleration abilities [73, 107, 108, 111]. In the study by Harper et al. [107], isokinetic concentric quadriceps and hamstring strength measured at faster (180°/s^−1^) velocities had large-to-very large associations with deceleration distance and time to stop, with the largest associations being reported in the non-dominant kicking leg. Greig and Naylor [111] also reported that isokinetic concentric quadriceps strength at faster velocities (180°/s^−1^) provided further predictive value (increase of 8%) of the deceleration distance to stop, in addition to various isokinetic eccentric hamstring strength qualities, during an unanticipated deceleration task in recreational RIMD sports players. Large associations have also been reported between concentric quadriceps strength at faster joint angular velocities (240°/s^−1^) and maximal horizontal braking power during a rapid horizontal deceleration [108]. These authors also reported that a rapid rate of concentric hamstring-to-quadriceps torque ratio, indicative of dynamic agonist–antagonist knee joint control, had large associations with maximal horizontal braking power. It was hypothesised that optimal balance between rapid hamstring-to-quadriceps torque would contribute to lower limb stiffness to enhance force attenuation, knee joint stability and force generation when braking hard during maximal horizontal decelerations. A significant increase in isokinetic concentric quadriceps strength of the left leg at 240°/s^−1^ was also observed following 6 weeks of speed and agility training with enforced decelerations, although this was not observed on the right leg, or in a group of players who followed the same speed and agility programme with no enforced decelerations [113]. Interestingly, in the same study, no significant changes in concentric hamstring strength at faster velocities (240°/s^−1^) were observed following each training programme. However, players who performed enforced decelerations had a significant increase in asymmetry in this NMP quality. Consequently, practitioners should be cognisant of the potential that deceleration training in individuals opting to brake harder, and more frequently, with a ‘preferred’ braking limb could promote greater inter-limb strength asymmetries. Thus, practitioners should train athletes to initiate and end horizontal deceleration manoeuvres with both limbs, and use other strength and conditioning modes to support the development and balance of these inter-limb NMP qualities. Whilst further research is required to investigate the transference of fast velocity concentric strength gains to rapid deceleration abilities, training interventions that can enhance fast velocity concentric strength qualities seem to be important for enhanced horizontal deceleration ability. In contrast, current research highlights a lower association between slower than higher angular velocity knee flexor and extensor concentric strength and horizontal deceleration performance. This is especially the case for the dominant leg in both male and female soccer and rugby players [107, 108, 113] and could be attributed to a lack of specificity to the fast joint angular velocities and muscle actions associated with rapid limb repositioning and braking during horizontal deceleration.

Greater concentric force production capabilities have also been reported in CMJ and DJ assessments in RIMD sport athletes categorised with high compared with low horizontal deceleration abilities [73]. However, it is important to note that these jump assessments are not isolated concentric actions, but rather stretch–shortening activities in which pre-impact muscle activations and eccentric muscle actions influence concentric phase performance and kinetics [114]. Indeed, Harper et al. [78] observed very large significant associations between DJ eccentric mean force and both concentric mean force (0.64–0.77) and GCT (− 0.73 to − 0.93), but only when measured from higher drop heights (i.e. 40 but not 20 cm). This suggests that eccentric mean force is important for greater concentric force and reduced GCT, likely owing to enhanced reflexes and a greater contribution of passive elastic structures in those capable of generating greater eccentric forces upon ground contact. Accordingly, developing greater eccentric force production capabilities appears to be an important pre-requisite for developing high concentric force production outputs in tasks that employ a rapid countermovement (i.e. stretch–shortening). These findings further support the importance of developing various eccentric force production and stretch–shortening qualities for generating the large braking forces necessary to rapidly reduce momentum, and for expanding a player’s repertoire of horizontal braking capabilities. It is also likely that greater lower limb strength facilitates reductions in eccentric work demands during eccentric braking activities, thereby facilitating dampening strategies that subsequently enhance deceleration efficiency [115].

### Rate of Force Development

The ability to pre-activate muscles to generate pre-tension prior to ground contact is an important NMP quality for enhancing horizontal deceleration ability especially when rapid adjustments of COM and high braking forces need to be generated in very short time frames (i.e. impulse), as illustrated in Figs. 1 and 2. Indeed, the NMP quality of ‘explosive’ strength (i.e. the ability to increase force production rapidly), as quantified by impulse during single-leg drop landing (at 25 ms) and isometric mid-thigh pull (at 100 and 300 ms) tests was associated with better performance in tasks with greater deceleration and braking demands [116, 117]. Similarly, both Behan et al. [118] and Jakobsen et al. [119] demonstrated the importance of isometric ankle plantar flexor rate of force development for enabling rapid adjustments and control of posture during highly dynamic sporting manoeuvres. This was suggested to be especially the case when a distal to proximal muscle activation sequence occurs, such as when braking during rapid horizontal decelerations. Accordingly, training interventions that enhance RFD, particularly of the ankle, seem especially important for enhancing horizontal deceleration ability, and should be investigated as a component of training interventions.

## Summary of Deterministic Factors Underpinning Horizontal Deceleration

**Fig. 4:**
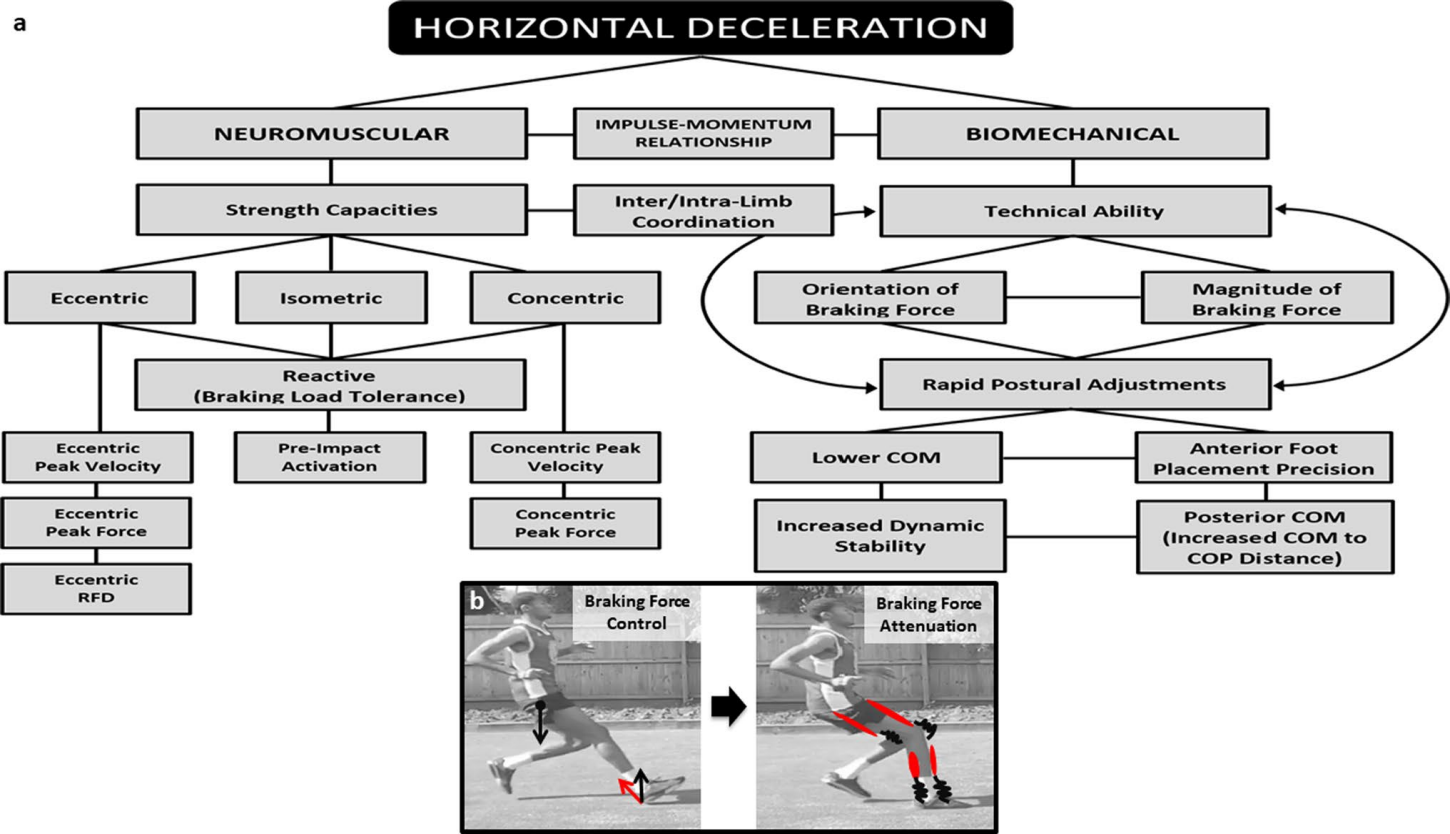
**a** Biomechanical and neuromuscular determinants of horizontal deceleration ability and **b** illustration of ‘braking force control’ and ‘braking force attenuation’ components. *COM* centre of mass, *COP* centre of pressure, *RFD* rate of force development

Using the previously identified biomechanical and neuromuscular determinants of horizontal deceleration, a summary of the deterministic factors is illustrated in Fig. 4a. The diagram displays horizontal deceleration ability as an interaction between the various neuromuscular and biomechanical qualities required to optimise braking impulse and to achieve the desired reductions in whole body momentum (i.e. reflecting the impulse-momentum relationship). This is underpinned by lower limb strength capacities and coordinative abilities that enable precise orientation and effectiveness of braking force application to be attained (i.e. large and rapid magnitude of braking force over a short time interval in the posterior direction), whilst proficiently distributing and attenuating impact forces throughout the lower limbs to minimise tissue damage.

**Fig. 5:**
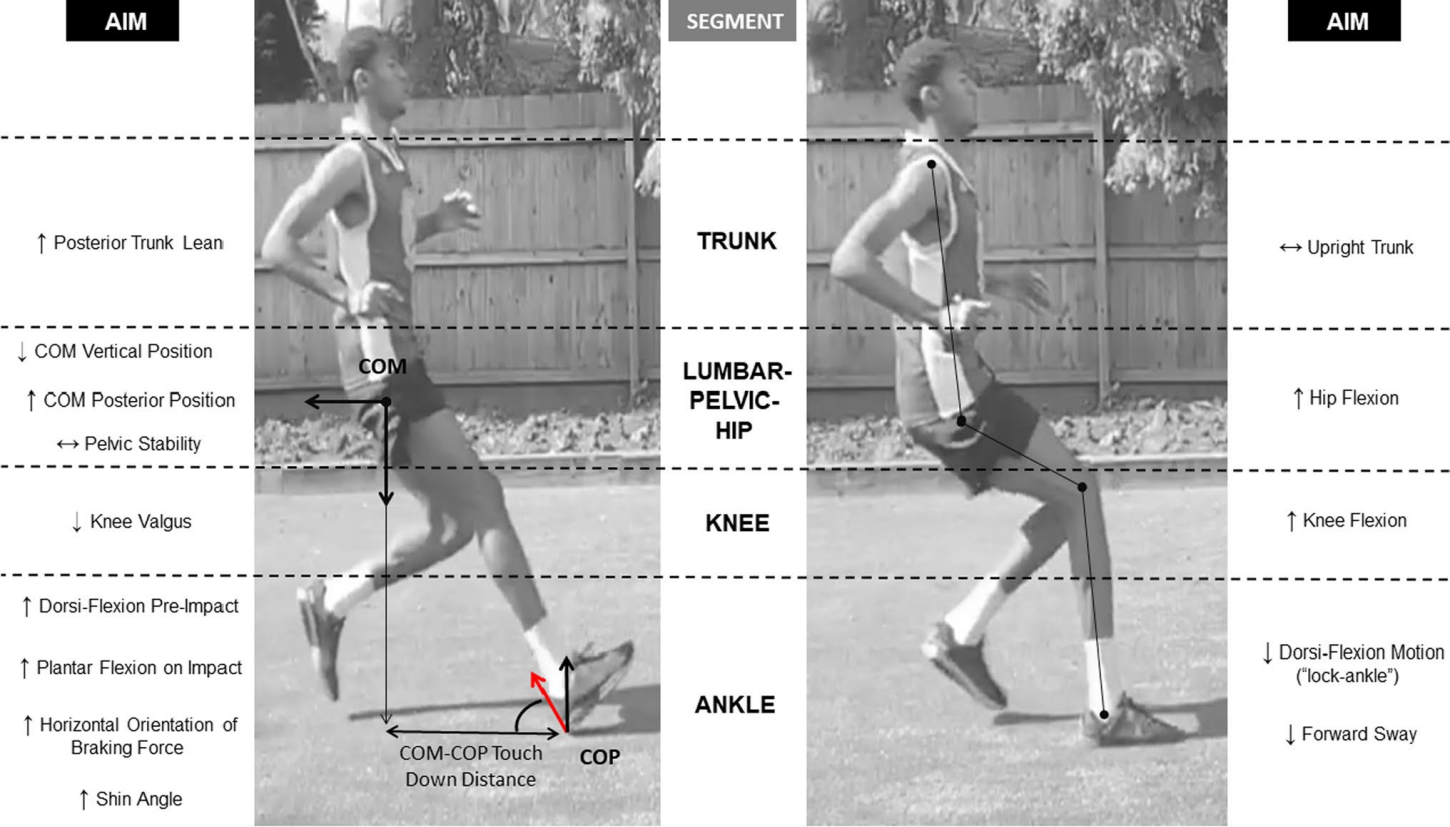
Kinematic factors underpinning maximal horizontal deceleration ability. *COM* centre of mass, *COP* centre of pressure, ↑ indicates increase, ↓ indicates decrease, ↔ indicates maintain/control

Based upon these considerations, we propose that horizontal deceleration ability could be defined as a “player’s ability to proficiently reduce whole body momentum, within the constraints, and in accordance with the specific objectives of the task (i.e. braking force control), whilst skilfully attenuating and distributing the forces associated with braking (i.e. braking force attenuation)”, these two key components are illustrated in Fig. 4b. Figure 5 provides further illustration of the key kinematic factors underpinning maximal horizontal deceleration ability.

## Limitations and Future Research Directions

Currently, the known biomechanical and NMP determinants of horizontal deceleration have been identified from cross-sectional observational studies. Subsequently, causality cannot be assumed. Nevertheless, these determinants provide a basis for the design of future research and training interventions. The ‘next step’ is to investigate if training interventions targeted to improve one or more of these underlying factors lead to improvements in horizontal deceleration ability. Potentially, there may also be a ‘ceiling’ value beyond which the key determinants of horizontal deceleration highlighted in this review provide no additional benefits to that ability. As such, the association between, and potential contribution of strength increases to horizontal deceleration ability would depend on the range of strength levels within the analysis, and the individual’s initial strength levels, respectively. This could be particularly relevant for higher performing athletes with elevated strength capacities. In such contexts, these athletes may require more highly focused training interventions that target specific horizontal deceleration deficiencies or NMP qualities that may better discriminate horizontal deceleration ability in higher level performers or those with a more extensive training history. This rationale supports the importance of methodologies to profile the muscles and NMP qualities underpinning horizontal deceleration ability. Critically, future research should investigate the degree to which improvements in various NMP qualities transfer to increases in horizontal deceleration ability (i.e. transference effect) across sexes and different standards of athletes.

Furthermore, current investigations have focused on understanding the importance of single-joint strength capacities and multi-joint assessments with a vertical force component. Accordingly, there is a need for future research to investigate the importance of horizontal force and power, particularly as these tests may possess greater specificity to the demands of braking during horizontal decelerations [120]. Motorised resistance devices, currently used more extensively for the assessment and training of horizontal acceleration and top speed capabilities [121–123], may provide useful applications here through means of assisted loads, which can augment braking demands during horizontal jumping and deceleration COD manoeuvres [124]. Another potential limitation of current research is that the determinants of horizontal deceleration have been identified mainly through performance in pre-planned horizontal deceleration tasks, with deceleration evaluated through whole body performance outcomes (e.g. distance to stop [metres], time to stop (seconds), average deceleration [m·s^−2^]). Accordingly, new technologies such as wearable sensors (e.g. inertial measurement units, foot force/pressure sensing insoles) and marker-less motion tracking combined with evolving machine learning algorithms may help to advance insights into the inter-limb braking demands of horizontal deceleration, alongside whole body deceleration performance outcomes [51, 125]. Given that horizontal decelerations during match play are largely unanticipated, future research should seek to determine how the importance of the biomechanical and neuromuscular determinants identified here for pre-planned horizontal deceleration ability might differ in their associations with un-anticipated horizontal deceleration ability. It also seems essential to gain further contextual information on why horizontal decelerations are performed during match play to better inform training prescription. Furthermore, the evident frequency, severity and importance of horizontal decelerations during RIMD sports highlight the importance of future research to further investigate the determinants of repeated horizontal deceleration ability. For example, what is the interaction between the ability to produce a one-off maximal horizontal deceleration and the ability to perform repeated intense horizontal decelerations? The latter may be particularly important because repeated intense horizontal decelerations are associated with muscle damage and residual decrements in various NMP qualities that could affect not only deceleration performance, but also performance in other important high-intensity sporting actions [126, 127].

Accordingly, our definition of horizontal deceleration identifies the potential importance of an individual’s ability to attenuate high-impact forces when braking, particularly during intense horizontal decelerations. We have also identified muscle groups that contribute to braking force attenuation, whilst also enabling higher braking force application. This raises some potentially important questions for future research that may have important implications for both performance and injury risk mitigation in RIMD sport athletes. For example, how do muscle–tendon units function during intense horizontal decelerations in different muscle groups? How do different muscle–tendon architectural properties enhance force attenuation capabilities during intense horizontal decelerations? Can different training methods enhance force attenuation capabilities and resistance to fatigue caused by repeated intense horizontal decelerations? Are different muscle fibre typologies better suited to meet the challenges of repeated intense decelerations in RIMD sports? Clearly, developing “new knowledge” in the areas identified in this review has the potential to improve player preparation for match play in a number of ways. First, by enhancing on-pitch performance through increased horizontal deceleration ability, which may also transfer to enhanced acceleration and COD abilities. Second, by increasing the capacity to perform horizontal decelerations more frequently and at a higher intensity. Finally, and ultimately, the improved horizontal deceleration ability may improve tissue tolerance and the ability to attenuate forces across both limbs leading to injury risk reduction. It is also important to note that the consequence of greater research on horizontal deceleration will be the development of a greater database of normative data that practitioners can use to compare their athlete’s horizontal deceleration performance, similar to what is available for horizontal acceleration. This may also provide a better understanding of the neuromuscular and biomechanical factors that may be less important or even detrimental to horizontal deceleration ability, in addition to how the importance of these factors may change for different population groups (e.g. sub-elite vs elite). We therefore take this opportunity to encourage future research into horizontal deceleration, and hope that this will lead to a more balanced understanding of how athletes slow down, in addition to speeding up!

## Conclusions

Recent information on match play movement demands in various RIMD sports illustrates that high-intensity decelerations are performed more frequently than equivalently intense accelerations. Intense horizontal decelerations require application of high braking forces in very short time periods to enable rapid reductions in whole body momentum. Similar to horizontal accelerations, horizontal decelerations require a highly co-ordinated sequence of lower-limb movements and rapid postural adjustments to achieve an effective application of braking force (i.e. braking technical ability). However, compared with other locomotor skills, horizontal decelerations have higher impact peaks and loading rates (i.e. tall thin impulse) that require an ability to attenuate forces efficiently throughout the lower limbs when braking. The increased demand to attenuate and distribute forces during horizontal decelerations places greater demand on eccentric muscle action, particularly during stances when muscles are trying to resist and control joint flexion. Accordingly, our review highlights that various eccentric strength qualities may contribute to improvements in both braking force application and attenuation capabilities. This review also illustrates that superior horizontal deceleration may be underpinned by isometric, concentric and reactive strength qualities, of which the latter may be an indicator of a player’s ability to pre-activate and pre-tension musculature prior to foot ground impact. Sports science and medicine practitioners can use the insights contained within this review to help prepare players for the horizontal deceleration demands of RIMD sports competition. We suggest a “mixed-method” approach, integrating traditional (local specificity) and coordinative (global specificity) training activities, is most likely to drive optimal adaptations across braking force application, braking force attenuation and overall horizontal deceleration abilities [128].

## References

[1] Harper DJ, Carling C, Kiely J (2019). High-intensity acceleration and deceleration demands in elite team sports competitive match play: a systematic review and meta-analysis of observational studies. Sport Med.

[2] Taylor JB, Wright AA, Dischiavi SL, Townsend MA, Marmon AR (2017). Activity demands during multi-directional team sports: a systematic review. Sport Med.

[3] Bellon CR, Deweese BH, Sato K, Clark KP, Stone MH (2019). Defining the early, mid, and late subsections of sprint acceleration in division I men’s soccer players. J Strength Cond Res.

[4] Nicholson B, Dinsdale A, Jones B, Till K (2021). The training of short distance sprint performance in football code athletes: a systematic review and meta-analysis. Sport Med.

[5] Buchheit M, Samozino P, Glynn JA, Michael BS, Al Haddad H, Mendez-Villanueva A (2014). Mechanical determinants of acceleration and maximal sprinting speed in highly trained young soccer players. J Sports Sci.

[6] Haugen TA, Breitschädel F, Seiler S (2020). Sprint mechanical properties in soccer players according to playing standard, position, age and sex. J Sports Sci.

[7] Haugen TA, Breitschädel F, Seiler S (2019). Sprint mechanical variables in elite athletes: are force-velocity profiles sport specific or individual?. PLoS ONE.

[8] Haugen TA, Breitschädel F, Seiler S (2019). Sprint mechanical properties in handball and basketball players. Int J Sports Physiol Perform.

[9] Jiménez-Reyes P, García-Ramos A, Cuadrado-Peñafiel V, Párraga-Montilla JA, Morcillo-Losa JA, Samozino P (2019). Differences in sprint mechanical force-velocity profile between trained soccer and futsal players. Int J Sports Physiol Perform.

[10] Morin JB, Gimenez P, Edouard P, Arnal P, Jiménez-Reyes P, Samozino P (2015). Sprint acceleration mechanics: the major role of hamstrings in horizontal force production. Front Physiol.

[11] Wild JJ, Bezodis IN, North JS, Bezodis NE (2018). Differences in step characteristics and linear kinematics between rugby players and sprinters during initial sprint acceleration. Eur J Sport Sci.

[12] Morris CG, Weber JA, Netto KJ (2022). Relationship between mechanical effectiveness in sprint running and force-velocity characteristics of a countermovement jump in Australian Rules Football athletes. J Strength Cond Res.

[13] Marcote-Pequeño R, García-Ramos A, Cuadrado-Peñafiel V, González-Hernández JM, Gómez MÁ, Jiménez-Reyes P (2019). Association between the force–velocity profile and performance variables obtained in jumping and sprinting in elite female soccer players. Int J Sports Physiol Perform.

[14] Hicks DS, Schuster JG, Samozino P, Morin J-B (2020). Improving mechanical effectiveness during sprint acceleration: practical recommendations and guidelines. Strength Cond J.

[15] Cahill MJ, Cronin JB, Oliver JL, Clark KP, Lloyd RS, Cross MR (2019). Sled pushing and pulling to enhance speed capability. Strength Cond J..

[16] Cahill MJ, Oliver JL, Cronin JB, Clark KP, Cross MR, Lloyd RS (2019). Sled-pull load–velocity profiling and implications for sprint training prescription in young male athletes. Sports.

[17] Cahill MJ, Oliver JL, Cronin JB, Clark KP, Cross MR, Lloyd RS (2020). Sled-push load-velocity profiling and implications for sprint training prescription in young athletes. J Strength Cond Res.

[18] Winkelman NC (2018). Attentional focus and cueing for speed development. Strength Cond J.

[19] Moir GL, Brimmer SM, Snyder BW, Connaboy C, Lamont HS (2018). Mechanical limitations to sprinting and biomechanical solutions: a constraints-led framework for the incorporation of resistance training to develop sprinting speed. Strength Cond J.

[20] Kovacs MS, Roetert EP, Ellenbecker TS (2008). Efficient deceleration: the forgotten factor in tennis-specific training. Strength Cond J.

[21] Dos’Santos T, Thomas C, Jones PA (2021). How early should you brake during a 180° turn? A kinetic comparison of the antepenultimate, penultimate, and final foot contacts during a 505 change of direction speed test. J Sports Sci.

[22] DosʼSantos T, Thomas C, Jones PA, Comfort P (2017). Mechanical determinants of faster change of direction speed performance in male athletes. J Strength Cond Res.

[23] Kaneko K, Hirano T, Yamagishi M, Kashiwagi Y, Hakamada N, Tago T (2019). Factors affecting the 180-degree change-of-direction speed in youth male soccer players. Hum Perform Meas.

[24] Harper DJ, Morin JB, Carling C, Kiely J (2020). Measuring maximal horizontal deceleration ability using radar technology: reliability and sensitivity of kinematic and kinetic variables. Sport Biomech.

[25] Graham-Smith P, Rumpf M, Jones PA. Assessment of deceleration ability and relationship to approach speed and eccentric strength. In: ISBS conference proc arch. 2018. p. 36.

[26] Oliva-Lozano JM, Fortes V, Krustrup P, Muyor JM (2020). Acceleration and sprint profiles of professional male football players in relation to playing position. PLoS ONE.

[27] Martínez Hernández D, Quinn M, Jones P (2022). Linear advancing actions followed by deceleration and turn are the most common movements preceding goals in male professional soccer. Sci Med Footb.

[28] Rhodes D, Valassakis S, Bortnik L, Eaves R, Harper D, Alexander J (2021). The effect of high-intensity accelerations and decelerations on match outcome of an elite English league two football team. Int J Environ Res Public Health.

[29] Vázquez-Guerrero J, Suarez-Arrones L, Gómez DC, Rodas G (2018). Comparing external total load, acceleration and deceleration outputs in elite basketball players across positions during match play. Kinesiology.

[30] Verheul J, Nedergaard NJ, Pogson M, Lisboa P, Gregson W, Vanrenterghem J (2021). Biomechanical loading during running: can a two mass-spring-damper model be used to evaluate ground reaction forces for high-intensity tasks?. Sport Biomech.

[31] Hader K, Mendez-Villanueva A, Palazzi D, Ahmaidi S, Buchheit M (2016). Metabolic power requirement of change of direction speed in young soccer players: not all is what it seems. PLoS ONE.

[32] Zamparo P, Pavei G, Monte A, Nardello F, Otsu T, Numazu N (2019). Mechanical work in shuttle running as a function of speed and distance: implications for power and efficiency. Hum Mov Sci.

[33] Gray A, Andrews M, Waldron M, Jenkins D. A model for calculating the mechanical demands of overground running. Sport Biomech. 2020;1–22. 10.1080/14763141.2020.1795238 32951525

[34] Hewit J, Cronin J, Button C, Hume P (2011). Understanding deceleration in sport. Strength Cond J.

[35] Alcazar J, Csapo R, Ara I, Alegre LM (2019). On the shape of the force-velocity relationship in skeletal muscles: the linear, the hyperbolic, and the double-hyperbolic. Front Physiol.

[36] Dalen T, Ingebrigtsen J, Ettema G, Hjelde GH, Wisløff U (2016). Player load, acceleration, and deceleration during forty-five competitive matches of elite soccer. J Strength Cond Res.

[37] Gastin PB, Hunkin SL, Fahrner B, Robertson S (2019). Deceleration, acceleration, and impacts are strong contributors to muscle damage in professional Australian football. J Strength Cond Res.

[38] de Hoyo M, Cohen DD, Sañudo B, Carrasco L, Álvarez-Mesa A, Del Ojo JJ (2016). Influence of football match time-motion parameters on recovery time course of muscle damage and jump ability. J Sports Sci.

[39] Russell M, Sparkes W, Northeast J, Cook CJ, Bracken RM, Kilduff LP (2016). Relationships between match activities and peak power output and creatine kinase responses to professional reserve team soccer match-play. Hum Mov Sci.

[40] Young WB, Hepner J, Robbins DW (2012). Movement demands in Australian rules football as indicators of muscle damage. J Strength Cond Res.

[41] Jaspers A, Kuyvenhoven JP, Staes F, Frencken WGP, Helsen WF, Brink MS (2018). Examination of the external and internal load indicators’ association with overuse injuries in professional soccer players. J Sci Med Sport.

[42] Bowen L, Gross AS, Gimpel M, Bruce-Low S, Li FX (2020). Spikes in acute:chronic workload ratio (ACWR) associated with a 5–7 times greater injury rate in English Premier League football players: a comprehensive 3-year study. Br J Sports Med.

[43] Della Villa F, Buckthorpe M, Grassi A, Nabiuzzi A, Tosarelli F, Zaffagnini S (2020). Systematic video analysis of ACL injuries in professional male football (soccer): injury mechanisms, situational patterns and biomechanics study on 134 consecutive cases. Br J Sports Med.

[44] Boden BP, Torg JS, Knowles SB, Hewett TE (2009). Video analysis of anterior cruciate ligament injury: Abnormalities in hip and ankle kinematics. Am J Sports Med.

[45] Johnston JT, Mandelbaum BR, Schub D, Rodeo SA, Matava MJ, Silvers-Granelli HJ (2018). Video analysis of anterior cruciate ligament tears in professional American football athletes. Am J Sports Med.

[46] Brophy RH, Stepan JG, Silvers HJ, Mandelbaum BR (2015). Defending puts the anterior cruciate ligament at risk during soccer: a gender-based analysis. Sports Health.

[47] Cochrane JL, Lloyd DG, Buttfield A, Seward H, McGivern J (2007). Characteristics of anterior cruciate ligament injuries in Australian football. J Sci Med Sport.

[48] Waldén M, Krosshaug T, Bjørneboe J, Andersen TE, Faul O, Hägglund M (2015). Three distinct mechanisms predominate in noncontact anterior cruciate ligament injuries in male professional football players: a systematic video analysis of 39 cases. Br J Sports Med.

[49] Varley MC, Fairweather IH, Aughey RJ (2012). Validity and reliability of GPS for measuring instantaneous velocity during acceleration, deceleration, and constant motion. J Sports Sci.

[50] Ade J, Fitzpatrick J, Bradley PS (2016). High-intensity efforts in elite soccer matches and associated movement patterns, technical skills and tactical actions: information for position-specific training drills. J Sports Sci.

[51] Harper DJ, Sandford GN, Clubb J, Young M, Taberner M, Rhodes D (2021). Elite football of 2030 will not be the same as that of 2020: what has evolved and what needs to evolve?. Scand J Med Sci Sport.

[52] Nassis GP, Massey A, Jacobsen P, Brito J, Randers MB, Castagna C (2020). Elite football of 2030 will not be the same as that of 2020: preparing players, coaches, and support staff for the evolution. Scand J Med Sci Sports.

[53] Griffin J, Horan S, Keogh J, Andreatta M, Minahan C (2021). Time to be negative about acceleration: a spotlight on female football players. J Strength Cond Res.

[54] McBurnie AJ, Harper DJ, Jones PA, Dos’Santos T (2022). Deceleration training in team sports: another potential “vaccine” for sports-related injury?. Sports Med.

[55] Dos’Santos T, Thomas C, Comfort P, Jones PA (2018). The effect of angle and velocity on change of direction biomechanics: an angle-velocity trade-off. Sport Med.

[56] Falch HN, Rædergård HG, van den Tillaar R (2019). Effect of different physical training forms on change of direction ability: a systematic review and meta-analysis. Sport Med Open.

[57] DosʼSantos T, Thomas C, Comfort P, Jones PA (2019). Role of the penultimate foot contact during change of direction: implications on performance and risk of injury. Strength Cond J.

[58] Verheul J, Warmenhoven J, Lisboa P, Gregson W, Vanrenterghem J, Robinson MA (2019). Identifying generalised segmental acceleration patterns that contribute to ground reaction force features across different running tasks. J Sci Med Sport.

[59] Lozano-Berges G, Clansey AC, Casajús JA, Lake MJ (2021). Lack of impact moderating movement adaptation when soccer players perform game specific tasks on a third-generation artificial surface without a cushioning underlay. Sport Biomech.

[60] Jordan AR, Carson HJ, Wilkie B, Harper DJ (2021). Validity of an inertial measurement unit system to assess lower-limb kinematics during a maximal linear deceleration. Cent Eur J Sport Sci Med.

[61] Nedergaard NJ, Kersting U, Lake M (2014). Using accelerometry to quantify deceleration during a high-intensity soccer turning manoeuvre. J Sports Sci.

[62] Dos’Santos T, Thomas C, Jones PA (2021). The effect of angle on change of direction biomechanics: comparison and inter-task relationships. J Sports Sci.

[63] Jones PA, Herrington L, Graham-Smith P (2016). Braking characteristics during cutting and pivoting in female soccer players. J Electromyogr Kinesiol.

[64] Jones PA, Herrington LC, Graham-Smith P (2016). Technique determinants of knee abduction moments during pivoting in female soccer players. Clin Biomech.

[65] Dos’Santos T, Thomas C, McBurnie A, Comfort P, Jones PA (2021). Biomechanical determinants of performance and injury risk during cutting: a performance-injury conflict?. Sport Med.

[66] Thomas C, Dos’Santos T, Comfort P, Jones PA (2020). Male and female soccer players exhibit different knee joint mechanics during pre-planned change of direction. Sport Biomech..

[67] Jones PA, Herrington LC, Graham-Smith P (2015). Technique determinants of knee joint loads during cutting in female soccer players. Hum Mov Sci.

[68] Thomas C, DosʼSantos T, Comfort P, Jones PA (2020). Effect of asymmetry on biomechanical characteristics during 180° change of direction. J Strength Cond Res.

[69] Clarke R, Read PJ, De Ste Croix MBA, Hughes JD (2020). The deceleration deficit: a novel field-based method to quantify deceleration during change of direction performance. J Strength Cond Res.

[70] Edwards WB (2018). Modeling overuse injuries in sport as a mechanical fatigue phenomenon. Exerc Sport Sci Rev.

[71] Hart NH, Nimphius S, Weber J, Spiteri T, Rantalainen T, Dobbin M (2016). Musculoskeletal asymmetry in football athletes: a product of limb function over time. Med Sci Sports Exerc.

[72] Morin JB, Samozino P (2016). Interpreting power-force-velocity profiles for individualized and specific training. Int J Sports Physiol Perform.

[73] Harper DJ, Cohen DD, Carling C, Kiely J (2020). Can countermovement jump neuromuscular performance qualities differentiate maximal horizontal deceleration ability in team sport athletes?. Sports (Basel).

[74] Herzog W (2018). Why are muscles strong, and why do they require little energy in eccentric action?. J Sport Health Sci.

[75] Falch HN, Kristiansen EL, Haugen ME, van den Tillaar R (2021). Association of performance in strength and plyometric tests with change of direction performance in young female team-sport athletes. J Funct Morphol Kinesiol.

[76] Jian Y, Winter D, Ishac M, Gilchrist L (1993). Trajectory of the body COG and COP during initiation and termination of gait. Gait Posture.

[77] Havens KL, Sigward SM (2015). Whole body mechanics differ among running and cutting maneuvers in skilled athletes. Gait Posture.

[78] Harper DJ, Cohen DD, Rhodes D, Carling C, Kiely J (2021). Drop jump neuromuscular performance qualities associated with maximal horizontal deceleration ability in team sport athletes. Eur J Sport Sci.

[79] Gageler WH, Thiel D, Neville J, James DA (2013). Feasibility of using virtual and body worn inertial sensors to detect whole-body decelerations during stopping. Proc Eng.

[80] Cohen DD, Restrepo A, Richter C, Harry JR, Franchi MV, Restrepo C (2021). Detraining of specific neuromuscular qualities in elite footballers during COVID-19 quarantine. Sci Med Footb.

[81] Mateus RB, Ferrer-Roca V, João F, Veloso AP (2020). Muscle contributions to maximal single-leg forward braking and backward acceleration in elite athletes. J Biomech.

[82] Colby S, Francisco A, Yu B, Kirkendall D, Finch M, Garrett W (2000). Electromyographic and kinematic analysis of cutting maneuvers implications for anterior cruciate ligament injury. Am J Sports Med.

[83] Mendiguchia J, Alentorn-Geli E, Idoate F, Myer GD (2013). Rectus femoris muscle injuries in football: a clinically relevant review of mechanisms of injury, risk factors and preventive strategies. Br J Sports Med.

[84] DosʼSantos T, McBurnie A, Thomas C, Comfort P, Jones PA (2020). Biomechanical determinants of the modified and traditional 505 change of direction speed test. J Strength Cond Res.

[85] Cesar GM, Sigward SM (2015). Dynamic stability during running gait termination: Differences in strategies between children and adults to control forward momentum. Hum Mov Sci.

[86] Lucarno S, Zago M, Buckthorpe M, Grassi A, Tosarelli F, Smith R (2021). Systematic video analysis of anterior cruciate ligament injuries in professional female soccer players. Am J Sports Med.

[87] Della Villa F, Tosarelli F, Ferrari R, Grassi A, Ciampone L, Nanni G (2021). Systematic video analysis of anterior cruciate ligament injuries in professional male rugby players: pattern, injury mechanism, and biomechanics in 57 consecutive cases. Orthop J Sport Med.

[88] Maniar N, Schache AG, Sritharan P, Opar DA (2018). Non-knee-spanning muscles contribute to tibiofemoral shear as well as valgus and rotational joint reaction moments during unanticipated sidestep cutting. Sci Rep.

[89] El-Ashker S, Chaabene H, Prieske O, Abdelkafy A, Ahmed MA, Muaidi QI (2019). Effects of neuromuscular fatigue on eccentric strength and electromechanical delay of the knee flexors: the role of training status. Front Physiol.

[90] Delextrat A, Piquet J, Matthews M, Cohen DD (2018). Strength-endurance training reduces the hamstrings strength decline following simulated football competition in female players. Front Physiol.

[91] Jones PA, Thomas C, Dos’Santos T, McMahon JJ, Graham-Smith P (2017). The role of eccentric strength in 180° turns in female soccer players. Sports (Basel)..

[92] Falch HN, Rædergård HG, van den Tillaar R (2020). Effect of approach distance and change of direction angles upon step and joint kinematics, peak muscle activation, and change of direction performance. Front Sport Act Living.

[93] Bishop MD, Brunt D, Pathare N, Patel B (2002). The interaction between leading and trailing limbs during stopping in humans. Neurosci Lett.

[94] Morin JB, Edouard P, Samozino P (2011). Technical ability of force application as a determinant factor of sprint performance. Med Sci Sports Exerc.

[95] Cesar GM, Sigward SM (2016). Dynamic stability during running gait termination: Predictors for successful control of forward momentum in children and adults. Hum Mov Sci.

[96] Hase K, Stein RB (1998). Analysis of rapid stopping during human walking. J Neurophysiol.

[97] Santoro E, Tessitore A, Liu C, Chen C-H, Khemtong C, Mandorino M (2021). The biomechanical characterization of the turning phase during a 180° change of direction. Int J Environ Res Public Health.

[98] Di Paolo S, Zaffagnini S, Tosarelli F, Aggio F, Bragonzoni L, Grassi A (2021). A 2D qualitative movement assessment of a deceleration task detects football players with high knee joint loading. Knee Surg Sports Traumatol Arthrosc.

[99] Straub RK, Horgan A, Powers CM (2021). Estimation of vertical ground reaction force parameters during athletic tasks using 2D video. Gait Posture.

[100] Graham-Smith P, Jones PA, Read P (2020). Taking a step back to reconsider change of direction and its application following ACL injury. Aspetar Sport Med J.

[101] Marques JB, Paul DJ, Graham-Smith P, Read PJ (2020). Change of direction assessment following anterior cruciate ligament reconstruction: a review of current practice and considerations to enhance practical application. Sport Med.

[102] Peel SA, Schroeder LE, Sievert ZA, Weinhandl JT (2019). Comparing anterior cruciate ligament injury risk variables between unanticipated cutting and decelerating tasks. J Appl Biomech.

[103] Dix C, Arundale A, Silvers-Granelli H, Marmon A, Zarzycki R, Snyder-Mackler L (2020). Biomechanical measures during two sport-specific tasks differentiate between soccer players who go on to anterior cruciate ligament injury and those who do not: a prospective cohort analysis. Int J Sports Phys Ther.

[104] Chaabene H, Prieske O, Negra Y, Granacher U (2018). Change of direction speed: toward a strength training approach with accentuated eccentric muscle actions. Sport Med.

[105] Dos’Santos T, Bishop C, Thomas C, Comfort P, Jones PA (2019). The effect of limb dominance on change of direction biomechanics: a systematic review of its importance for injury risk. Phys Ther Sport.

[106] Donelon TA, Dos’Santos T, Pitchers G, Brown M, Jones PA (2020). Biomechanical determinants of knee joint loads associated with increased anterior cruciate ligament loading during cutting: a systematic review and technical framework. Sport Med Open..

[107] Harper DJ, Jordan AR, Kiely J (2021). Relationships between eccentric and concentric knee strength capacities and maximal linear deceleration ability in male academy soccer players. J Strength Cond Res.

[108] Zhang Q, Léam A, Fouré A, Wong DP, Hautier CA (2021). Relationship between explosive strength capacity of the knee muscles and deceleration performance in female professional soccer players. Front Physiol.

[109] Van Hooren B, Zolotarjova J (2017). The difference between countermovement and squat jump performances: a review of underlying mechanisms with practical applications. J Strength Cond Res.

[110] Bogdanis GC, Tsoukos A, Brown LE, Selima E, Veligekas P, Spengos K (2018). Muscle fiber and performance changes after fast eccentric complex training. Med Sci Sports Exerc.

[111] Greig M, Naylor J (2017). The efficiacy of angle-matched isokinetic knee flexor and extensor strength parameters in predicting agility test performance. Int J Sports Phys Ther.

[112] Douglas J, Pearson S, Ross A, McGuigan M (2020). Reactive and eccentric strength contribute to stiffness regulation during maximum velocity sprinting in team sport athletes and highly trained sprinters. J Sports Sci.

[113] Lockie RG, Schultz AB, Callaghan SJ, Jeffriess MD (2014). The effects of traditional and enforced stopping speed and agility training on multidirectional speed and athletic function. J Strength Cond Res.

[114] McBride JM, McCaulley GO, Cormie P (2008). Influence of preactivity and eccentric muscle activity on concentric performance during vertical jumping. J Strength Cond Res.

[115] McBride JM, Nimphius S (2020). Biological system energy algorithm reflected in sub-system joint work distribution movement strategies: influence of strength and eccentric loading. Sci Rep.

[116] Welch N, Richter C, Franklyn-Miller A, Moran K (2021). Principal component analysis of the biomechanical factors associated with performance during cutting. J Strength Cond Res.

[117] Thomas C, Comfort P, Chiang C, Jones PA (2015). Relationship between isometric mid-thigh pull variables and sprint and change of direction performance in collegiate athletes. J Trainol.

[118] Behan FP, Pain MTG, Folland JP (2018). Explosive voluntary torque is related to whole-body response to unexpected perturbations. J Biomech.

[119] Jakobsen MD, Sundstrup E, Krustrup P, Aagaard P (2011). The effect of recreational soccer training and running on postural balance in untrained men. Eur J Appl Physiol.

[120] Cronin JB, Ross A, Bedford C, Crosland R, Birch W, Fathers S (2016). Deceleration forces associated with a novel cable pulling exercise. J Aust Strength Cond.

[121] Lahti J, Jiménez-Reyes P, Cross MR, Samozino P, Chassaing P, Simond-Cote B (2020). Individual sprint force-velocity profile adaptations to in-season assisted and resisted velocity-based training in professional rugby. Sports.

[122] Cross MR, Lahti J, Brown SR, Chedati M, Jimenez-Reyes P, Samozino P (2018). Training at maximal power in resisted sprinting: optimal load determination methodology and pilot results in team sport athletes. PLoS ONE.

[123] Rakovic E, Paulsen G, Helland C, Eriksrud O, Haugen T (2018). The effect of individualised sprint training in elite female team sport athletes: a pilot study. J Sports Sci.

[124] Eriksrud O, Ahlbeck F, Harper DJ, Gløersen ØN (2022). Validity of velocity measurements of a motorized resistance device during change of direction. Front Physiol.

[125] Lloyd D (2021). The future of in-field sports biomechanics: wearables plus modelling compute real-time in vivo tissue loading to prevent and repair musculoskeletal injuries. Sport Biomech.

[126] Harper DJ, Kiely J (2018). Damaging nature of decelerations: do we adequately prepare players?. BMJ Open Sport Exerc Med.

[127] Lakomy J, Haydon DT (2004). The effects of enforced, rapid deceleration on performance in a multiple sprint test. J Strength Cond Res.

[128] Brearley S, Bishop C (2019). Transfer of training: how specific should we be?. Strength Cond J.

